# The latent reservoir of inducible, infectious HIV-1 does not decrease despite decades of antiretroviral therapy

**DOI:** 10.1172/JCI171554

**Published:** 2023-09-01

**Authors:** Natalie F. McMyn, Joseph Varriale, Emily J. Fray, Carolin Zitzmann, Hannah MacLeod, Jun Lai, Anushka Singhal, Milica Moskovljevic, Mauro A. Garcia, Brianna M. Lopez, Vivek Hariharan, Kyle Rhodehouse, Kenneth Lynn, Pablo Tebas, Karam Mounzer, Luis J. Montaner, Erika Benko, Colin Kovacs, Rebecca Hoh, Francesco R. Simonetti, Gregory M. Laird, Steven G. Deeks, Ruy M. Ribeiro, Alan S. Perelson, Robert F. Siliciano, Janet M. Siliciano

**Affiliations:** 1 Johns Hopkins University School of Medicine, Baltimore, Maryland, USA.; 2 Los Alamos National Laboratory, Los Alamos, New Mexico, USA.; 3 AccelevirDx, Baltimore, Maryland, USA.; 4 The Wistar Institute, Philadelphia, Pennsylvania, USA.; 5 University of Pennsylvania, Philadelphia, Pennsylvania, USA.; 6 Philadelphia Field Initiating Group for HIV-1 Trials, Philadelphia, Pennsylvania, USA.; 7 Maple Leaf Medical Clinic, Toronto, Ontario, Canada.; 8 UCSF, San Francisco, California, USA.; 9 Howard Hughes Medical Institute, Baltimore, Maryland, USA.

**Keywords:** AIDS/HIV, Virology, Molecular biology, T cells

## Abstract

HIV-1 persists in a latent reservoir in resting CD4^+^ T cells despite antiretroviral therapy (ART). The reservoir decays slowly over the first 7 years of ART (*t*_1/2_ = 44 months). However, whether decay continues with long-term ART is unclear. Recent integration site studies indicate gradual selection against inducible, intact proviruses, raising speculation that decades of ART might allow treatment interruption without viral rebound. Therefore, we measured the reservoir in 42 people on long-term ART (mean 22 years) using a quantitative viral outgrowth assay. After 7 years of ART, there was no long-term decrease in the frequency of inducible, replication-competent proviruses but rather an increase with an estimated doubling time of 23 years. Another reservoir assay, the intact proviral DNA assay, confirmed that reservoir decay with *t*_1/2_ of 44 months did not continue with long-term ART. The lack of decay reflected proliferation of infected cells. Most inducible, replication-competent viruses (79.8%) had *env* sequences identical to those of other isolates from the same sample. Thus, although integration site analysis indicates changes in reservoir composition, the proliferation of CD4^+^ T cells counteracts decay, maintaining the frequency of inducible, replication-competent proviruses at roughly constant levels over the long term. These results reinforce the need for lifelong ART.

## Graphical abstract

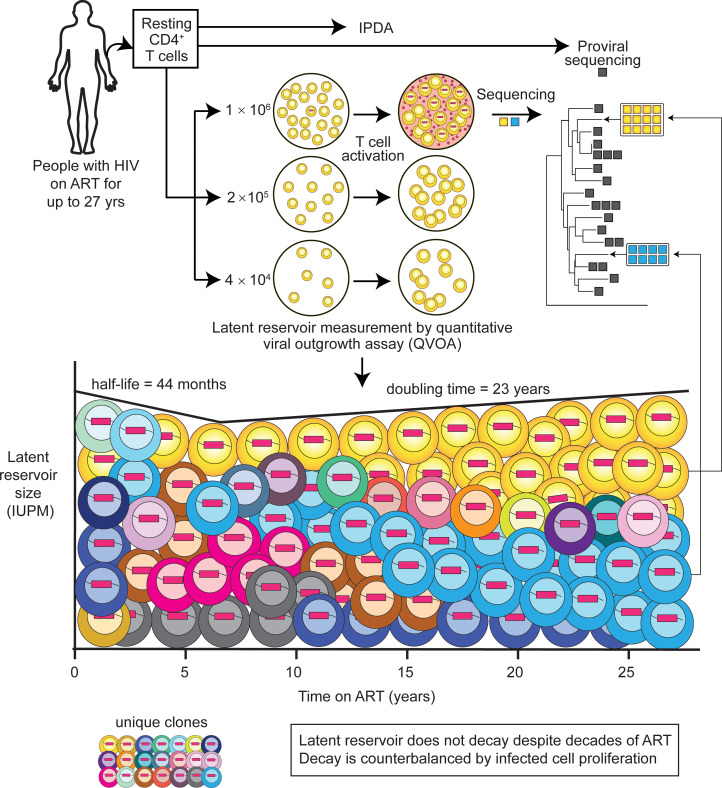

## Introduction

Antiretroviral therapy (ART) blocks HIV-1 replication, reducing plasma virus levels to below the detection limit of clinical assays (1–3). ART prevents disease progression but does not eliminate the latent reservoir, a population of CD4^+^ T cells carrying transcriptionally silent but potentially inducible replication-competent proviruses (4–9). This pool of latent virus is established early in infection (10, 11) and resides in resting CD4^+^ T cells, which are relatively non-permissive for viral gene expression (12–19) owing to the lack of active nuclear forms of required host transcription factors, including NF-κB and NFAT (12, 13, 16), as well as the elongation factor P-TEFb (refs. 15, 17–19, and reviewed in ref. 20). The lack of viral gene expression enables the cells to escape viral cytopathic effects and host immune clearance. HIV-1 cure strategies aim to eliminate the latent, replication-competent proviruses that are responsible for viral rebound when treatment is interrupted (21–23). Agents that reverse latency may allow killing of infected cells (24–26). An alternative approach is the induction of immune control without reservoir elimination (25, 27, 28).

The latent reservoir is stable in people with HIV (PWH) on ART. Early studies during the first 3 years of ART defined a half-life of 44.2 months (9). This half-life was confirmed in a follow-up study based on 7 years of ART (29) and reconfirmed in PWH on newer regimens that include integrase inhibitors (30). These studies used the quantitative viral outgrowth assay (QVOA), a limiting-dilution virus culture assay in which mitogen-induced T cell activation reverses latency and allows exponential viral outgrowth from individual latently infected cells (31, 32). The QVOA provides a definitive minimal estimate of the frequency of cells carrying inducible, replication-competent proviruses. Reservoir decay has also been studied using the intact proviral DNA assay (IPDA), a digital droplet PCR assay that interrogates individual proviruses to distinguish intact proviruses from the excess of defective proviruses present in treated PWH (33–36). The IPDA excludes most but not all defective proviruses and provides a maximal estimate of the frequency of cells harboring intact proviruses (34, 37). During the first several years of ART, the frequency of cells with intact proviruses decreases at a rate similar to that measured with the QVOA. Interestingly, defective proviruses showed no decay over the same time interval (35, 36, 38). These results raised the possibility that some selective pressure operates on cells with intact proviruses. One large-scale IPDA study found reservoir decay with a half-life of 48 months for the first 7 years of ART. Subsequently, decay slowed substantially (half-life = 224.4 months) (35). Variable patterns of long-term decay were seen in individual participants in another study (39).

The IPDA provides a maximal estimate of reservoir size but does not provide information on the clonality and inducibility of latent proviruses, factors critical for understanding HIV-1 persistence. The long half-life of the latent HIV-1 reservoir can be explained by the balance between cell loss and cell proliferation (40–48). Although the latent reservoir was originally demonstrated in resting CD4^+^ T cells (4, 6), it is now clear that most cells in the reservoir have some history of proliferation (41, 43, 44). Clonal expansion of HIV-1–infected cells is largely due to antigen-driven proliferation (49–52), but homeostatic proliferation (53) and effects related to the site of integration (43, 44) also contribute. As a result of proliferation, over 50% of independent isolates of replication-competent virus from a single blood sample share sequence identity with other isolates from the same sample (45–47). Large, infected T cell clones carrying inducible, replication-competent proviruses can give rise to non-suppressible viremia in some PWH (54, 55). Certain infected cell clones expand and contract over time (56), making the reservoir highly dynamic. Upon treatment interruption, viral rebound may originate from large, expanded clones as well as minor variants (57–60).

There is substantial interest in how the inducibility of latent proviruses is impacted by the site of integration. Most HIV-1 integration sites are in actively transcribed host genes (61, 62). In expanded clones, integration sites are often found in a small number of genes related to cell survival or proliferation, suggesting a role for aberrant expression of those genes in clonal expansion, either directly or in synergy with antigen (43, 44, 63). Interesting recent studies in PWH who control HIV-1 replication without ART have shown that integration sites are predominantly in chromosomal regions associated with reduced transcription (64, 65). Similarly, in ART-treated individuals, expanded clones of infected cells carrying intact proviruses are frequently found in zinc finger genes associated with repressive chromatin marks (65, 66). Another study tracked proviral frequency and integration sites over a decade of ART and identified large transcriptionally active clones in active chromosomal regions (67). However, the authors suggested that these clones are an exception to a general trend for a longitudinal increase in intact proviruses integrated into non-genic or satellite DNA regions associated with lower transcriptional activity (67). In PWH on very long-term ART (20 years), surviving intact proviral clones were generally in chromosomal locations associated with reduced gene expression (68). These studies did not directly determine whether these proviruses could contribute to viral rebound. However, this work suggests that immune selection operating over long time intervals gives rise to latent reservoirs dominated by clones that are not readily induced due to their sites of integration, a suggestion with implications for a functional cure. Therefore, it is important to validate whether inducible, replication-competent proviruses decline significantly in PWH on very long-term ART with reservoir assays that evaluate whether proviruses can be induced.

Here, we use the QVOA, the IPDA, and proviral sequencing to evaluate the latent reservoir of 42 PWH who had maintained suppression of viral replication on ART for 14–27 years to determine whether the reservoir remains a barrier to cure.

## Results

### Study participants.

Through discussions with care providers at HIV specialty clinics in Baltimore, San Francisco, Philadelphia, and Toronto, we identified 42 PWH who had maintained suppression of viremia on very long-term ART (Supplemental Table 1). The average time between the start of a continuously suppressive ART regimen and the most recent sample date was 22.1 years (range 14.9–26.8 years). Demographics of the study population (88% male, 57% White, 33% African American, 10% other) reflect the participating clinics. Participants started suppressive ART at a mean age of 40.8 years. The mean nadir CD4^+^ T cell count was 161 cells/μL, and the mean age at the last sample time was 63 years.

**Figure 1:**
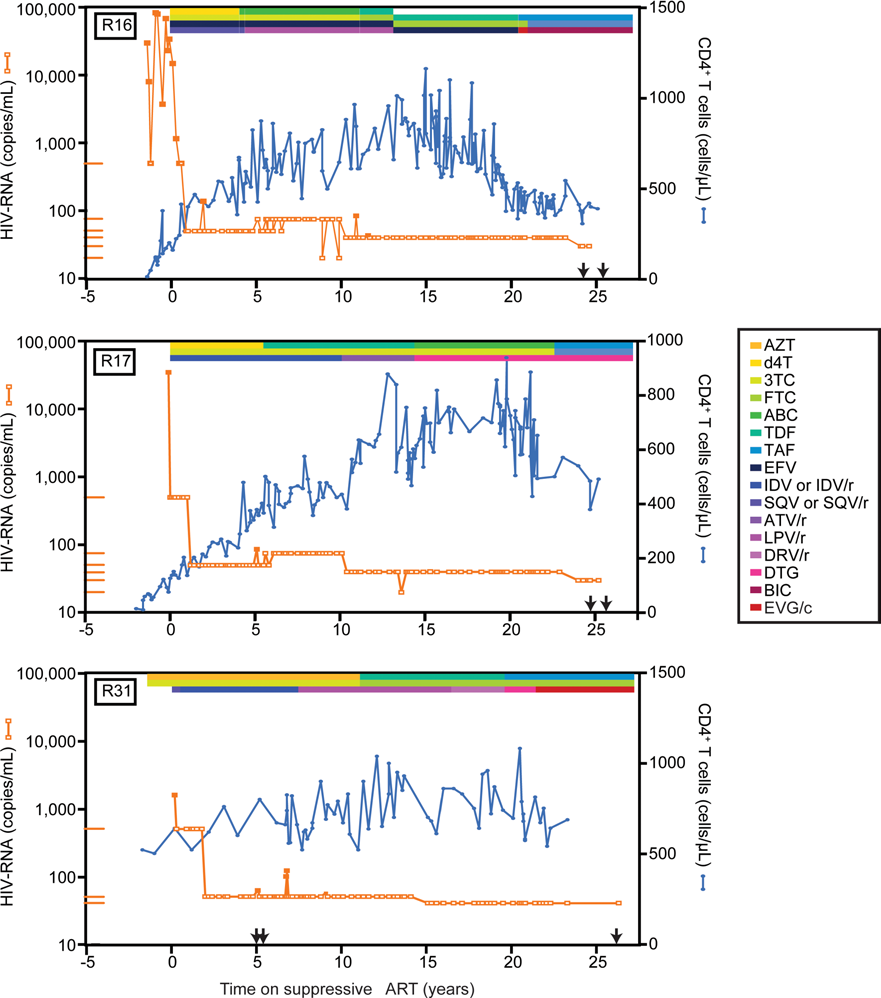
Plasma HIV-1 RNA levels and CD4^+^ T cell counts of representative participants R16, R17, and R31, who had been on suppressive ART for 25 years. Levels of plasma HIV-1 RNA in copies/mL (orange squares) and CD4^+^ T cells in cells/μL (blue circles) are plotted as a function of time on suppressive ART. Plasma HIV-1 RNA values below the limit of detection are denoted by open orange squares and are plotted at the limit of detection of the assay used. The limits of detection of assays used at various times are shown by orange lines along the *y* axis. The ART regimens are given by the colored bars at the top of each plot. Times of sampling for reservoir assays are indicated (black arrows). Pre-ART plasma HIV-1 RNA levels for R31 were not available. The first available measurement was obtained while the participant was taking azidothymidine and lamivudine.

Plasma HIV-1 RNA measurements and CD4^+^ T cell counts for 3 representative participants who had been on suppressive ART for approximately 25 years are shown in Figure 1. Clinical data for other representative participants are given in Supplemental Figures 1–3 and Supplemental Tables 1–3. Isolated single plasma HIV-1 RNA measurements above the detection limit (“blips”) were not considered an exclusion because they typically reflect slight transient increases in the residual viremia present in all treated PWH and not non-adherence or treatment failure (69, 70). Study participants had an average of 73 viral load measurements each over the period of suppression. The limit of detection of the assay for plasma HIV-1 RNA was 400 copies/mL for some early measurements and 20 or 50 copies/mL for more recent measurements. Remarkably, of 3,018 total plasma HIV-1 RNA measurements in these participants during the period of suppression, only 3 measurements were greater than 400 copies/mL (Supplemental Table 3). One, a blip of 15,228 copies/mL for participant R23, was assumed to be a sample or laboratory error because a repeat measurement 18 days later was below 50 copies/mL. Similarly, blips of 3,851 and 1,030 copies/mL in participants R12 and R13 were followed by negative viral load measurements 7 and 13 days later, respectively. In all, 17 of 42 participants had blips greater than 100 copies/mL. Of 3,018 measurements of plasma HIV-1 RNA, only 24 (0.79%) were between 100 and 400 copies/mL (median 141 copies/mL; Supplemental Table 3). The level of plasma HIV-1 RNA returned to below the limit of detection on subsequent visits without regimen change (Supplemental Table 3). Blips generally occurred relatively early in the course of treatment (median 3.3 years), reflecting in part issues with earlier versions of the plasma HIV-1 RNA assay. In 2005, we showed that blips of less than 200 copies/mL were often not concordant on independent testing and were not associated with new resistance mutations (69). Thus, blips represent biological and statistical variation around mean HIV-1 levels below 50 copies/mL rather than clinically meaningful elevations in viremia. All participants had plasma HIV-1 RNA levels below the limit of detection (<20–40 copies/mL) at the time of sample collection. We conclude that the study population is a set of PWH who have maintained exceptional control of viremia on ART for very long periods of time.

### Resting CD4^+^ T cells carrying inducible, replication-competent proviruses are readily detected in PWH on very long-term ART.

It has been unclear whether the reservoir decay observed during the first several years of ART continues. Therefore, we measured the frequency of latently infected cells in 42 PWH who had maintained suppression of viremia on very long-term ART using the QVOA. Resting CD4^+^ T cells were purified from large-volume peripheral blood samples or leukapheresis products, resulting in a combined total of over 984 million resting CD4^+^ T cells assayed. For 18 of the 42 participants, longitudinal analysis was possible by repeated recent sampling and/or inclusion of previous QVOA measurements by our laboratory on samples from the same donors (29, 47, 56, 71, 72). All QVOA measurements were made using exactly the same protocol except for minor modifications previously shown to give the same infected-cell frequencies (73). Remarkably, 4 participants from the 2003 study of reservoir decay during the first 7 years of ART (29) had maintained long-term suppression of viremia and were included.

**Figure 2:**
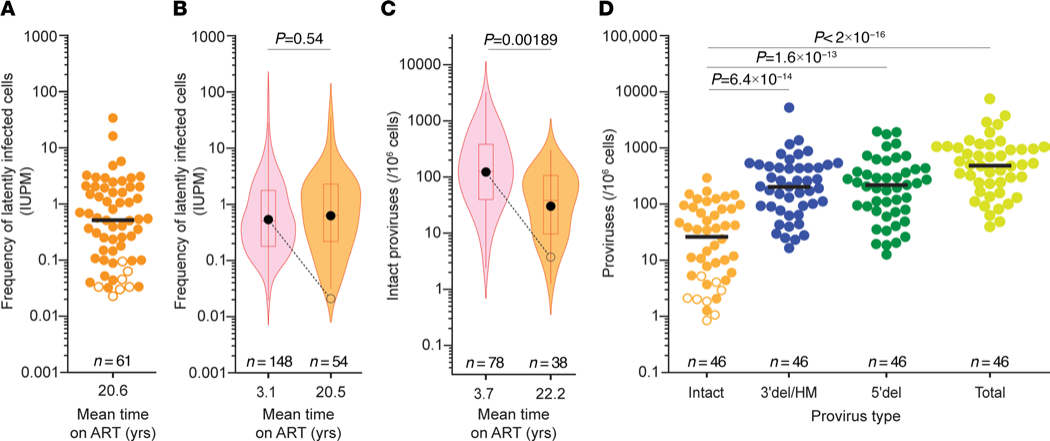
Reservoir measurements in PWH on long-term ART. (**A**) QVOA measurements of inducible, replication-competent proviruses among resting CD4^+^ T cells of PWH on ART for more than 7 years (mean 20.6 years). Virus was isolated in 54 of 61 assays from 42 participants. For negative assays (open symbols), a posterior median estimate is given (88). Black line, median value; *n*, number of assays. (**B**) Violin plots of the frequencies of latently infected cells in positive QVOAs on resting CD4^+^ T cells from 59 PWH on short-term ART (pink) and 38 PWH on long-term ART (orange). Mean duration of suppressive ART at sampling is on the *x* axis. Boxes and whiskers indicate middle quartiles and maximum and minimum values, respectively. Red horizontal lines, median; black circles, geometric mean. Expected decay with *t*_1/2_ = 44.2 months over the relevant time interval is indicated (dashed line). Short-term ART data are from the 2003 study (29). Short- and long-term frequencies were not statistically different (*P* = 0.54) by Student’s *t* test on log-transformed values. (**C**) Violin plots of the frequencies of intact proviruses as measured in positive IPDAs on samples from 62 PWH on short-term ART (pink) and 34 PWH on long-term ART (orange). Short-term ART data are from ref. 35 using samples obtained between 0.5 and 7 years after ART initiation. Expected decay with *t*_1/2_ = 44.2 months over the relevant time interval is indicated (dashed line). Log-transformed values were compared using Student’s *t* test. (**D**) IPDA measurements of the frequency of intact and defective proviruses (3′ deleted/hypermutated and 5′ deleted) in resting CD4^+^ T cells of PWH on suppressive ART for more than 7 years (mean = 22.0 years). Bars indicate median values. For negative assays (open symbols), maximum value based on the number of cells plated is shown. Significance values for multiple comparisons with the log-transformed frequencies of intact proviruses were determined using Dunnett’s test. *n*, number of assays.

Exponential viral outgrowth was detected in 54 of 61 assays on samples obtained after more than 7 years of ART (mean 20.6 years; Figure 2A). Because of the low frequency of latently infected cells, the number of purified resting CD4^+^ T cells obtained from some donors was insufficient to detect outgrowth. Only 7 of 61 assays (11.5%) were negative. This fraction is smaller than the fraction of negative assays in the original study of PWH on ART for less than 7 years (13.5%) (29). In most cases in which the QVOA was negative, the reservoir was also shown to be small by other assays (see below). The frequency distributions of resting CD4^+^ T cells carrying inducible, replication-competent proviruses as detected by the QVOA are shown in Figure 2B for the long-term ART cohort and for PWH in the original study (short-term cohort, ART for <7 years). The geometric mean frequency for positive assays in the long-term cohort was 0.62 infectious units per million (IUPM) resting CD4^+^ T cells, slightly higher than the geometric mean frequency for positive assays for the short-term cohort (0.54 IUPM; Figure 2B and Supplemental Table 4). This difference was not significant. This analysis includes multiple assays for some participants in each cohort. Using only the latest time point for each participant within each data set, we also found a slightly higher geometric mean frequency for the long-term cohort (0.53 vs. 0.45 IUPM), but the difference was not significant (*P* = 0.605 by Student’s *t* test on log-transformed values). The most important finding is that frequencies had not declined at the expected rate in PWH on very long-term ART. From a geometric mean value of 0.54 IUPM, decay with a half-life of 44.2 months over the long interval between the mean sampling times for the short- and long-term cohorts would give a frequency of only 0.02 IUPM (dashed line, Figure 2B), much lower than the observed frequency (0.62 IUPM; Figure 2B). Thus, our results suggest that the reservoir decay apparent over the first 7 years of ART (29, 30, 35) does not continue or is counterbalanced by another process that increases the frequency of latently infected cells.

### Intact proviruses are readily detectable in PWH on very long-term ART.

To confirm that reservoir decay with a half-life of 44.2 months does not continue with prolonged treatment, we used the IPDA (34) to measure the frequency of genetically intact and defective proviruses in resting CD4^+^ T cells from the same 42 PWH (Figure 2, C and D). Intact proviruses were detected in 38 of 46 assays (82.6%), a slightly lower fraction than observed in a previous study of PWH on ART for less than 7 years (91.8%; ref. 35). For positive assays, the geometric mean frequency of intact proviruses for the long-term cohort was 30.3 per 10^6^ resting CD4^+^ T cells (Figure 2, C and D). Because samples from the 2003 study (29) were not available for IPDA analysis, we compared the intact proviral frequencies in PWH on very long-term ART with those measured in a recent longitudinal analysis by Peluso et al. (35). For samples obtained from donors on ART for less than 7 years (mean 3.7 years), the geometric mean frequency of intact proviruses in positive assays was 122.7 per 10^6^ CD4^+^ T cells (Figure 2C). This value is significantly higher than the value measured in PWH on very long-term ART (30.3 per 10^6^ CD4^+^ T cells, *P* < 0.00189), indicating that there is some slow decay of intact proviruses over a long time interval. However, as shown in Figure 2C, the difference is much less than expected based on decay with a half-life of 44.2 months. The finding that very slow reservoir decay is detected with the IPDA but not the QVOA is discussed below. Importantly, both assays show that the decay of genetically intact proviruses does not continue with a half-life of 44.2 months in PWH on very long-term ART.

Intact proviruses constituted less than 10% of all proviruses detected in the IPDA (Figure 2D), consistent with other studies (34, 37). The median frequencies of different classes of proviruses per 10^6^ resting CD4^+^ T cells (intact, 26; 3′ defective, 203; 5′ defective, 213; and total calculated on a per-assay basis, 481) were slightly lower than those measured in CD4^+^ T cells in a large-scale IPDA analysis of PWH on ART for an average of 7.2 years (intact, 54; 3′ defective, 322; 5′ defective, 269; and total, 755; *n* = 400) (37). The preponderance of defective sequences was confirmed with full genome sequencing (see below). Consistent with previous studies (34, 74), the frequency of intact proviruses in PWH on very long-term ART was substantially (~50 times) higher than the frequency of latently infected cells detected in QVOA analysis of the same samples. This difference in part reflects the fact that only a fraction of intact proviruses is induced by a single round of T cell activation in the QVOA (33, 34, 74).

We observed a significant correlation between the QVOA and IPDA measurements for individual samples from the long-term cohort (*r* = 0.47, *P* = 0.0013; Supplemental Figure 4), consistent with previous studies (34). Four of five samples for which the QVOA failed to yield outgrowth also had low reservoir size by IPDA (Supplemental Figure 4), suggesting that failure to detect outgrowth in some samples is generally due to low overall reservoir size rather than low inducibility.

Together, these results demonstrate that proviruses with the potential to cause rebound are readily detectable in PWH on very long-term ART and do not continue to decay at rates observed during the initial years of treatment.

### After 7 years of ART, the frequency of cells with inducible, replication-competent proviruses may begin to increase.

**Figure 3:**
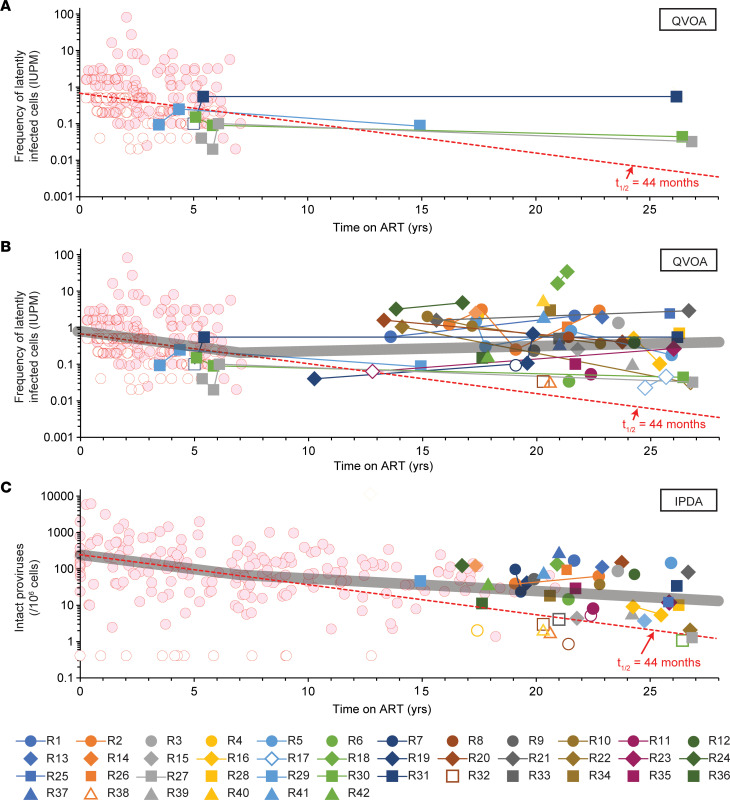
Decay of the latent reservoir during very long-term ART. (**A**) QVOA measurements of the frequencies of resting CD4^+^ T cells with inducible, replication-competent proviruses in 4 PWH from the original 2003 study (29) who were sampled again after a total of 15–27 years on suppressive ART. Frequencies for the first 7 years of ART from other participants in Siliciano et al. (29) are shown by red circles. For assays with no outgrowth, the median posterior estimate of infected-cell frequency based on input cell number was plotted (open symbols). Monophasic decay with *t*_1/2_ = 44 months is indicated by the dashed red line. (**B**) QVOA measurements for all participants in the current study. Lines connect longitudinal measurements in the same donor. A best fit model for segmented exponential decay (thick gray line) shows initial decay with *t*_1/2_ = 44 months followed by an inflection at 7 years and then a slow increase (doubling time = 23 years). See Supplemental Table 5 for details. (**C**) IPDA measurements of the decay of intact proviruses in PWH on very long-term ART. Frequencies for the initial years of ART (red circles) were replotted from Peluso et al. (35). Results are expressed as the DNA shearing index–corrected (DSI-corrected) geometric mean frequency of intact proviruses in resting CD4^+^ T cells (34). For assays in which no intact proviruses were detected, results are reported as the inverse of the number of cell equivalents analyzed (open symbols). The DSI was below 0.5 for all participants. Monophasic decay with a half-life of 44.2 months is indicated by the dashed line. A model for segmented exponential decay (thick gray line) shows initial decay with *t*_1/2_ = 46 months followed by an inflection at 7 years and slower subsequent decay (*t*_1/2_ = 9 years). See Supplemental Table 6 for details.

To confirm that reservoir decay observed during the first 7 years of ART (*t*_1/2_ = 44.2 months) (29, 30) does not continue, we plotted the frequency of resting CD4^+^ T cells with inducible, replication-competent virus as a function of time on suppressive ART (Figure 3, A and B). Data from the 2003 study (29) were also plotted to show reservoir decay kinetics in the early years of ART. For 4 participants (R27, R29, R30, and R31) who were part of the 2003 study (29), there was virtually no change between measurements made in the initial study and measurements made after 15–27 years of ART (Figure 3A). QVOA data for all participants in the long-term cohort (Figure 3B) clearly show that after 7 years, reservoir decay does not continue. All positive assays after 13 years of ART show a higher frequency than expected for decay at *t*_1/2_ = 44.2 months from a starting frequency of 1 IUPM.

We used a mixed-effects modeling approach to fit different models to the long-term decay data (Supplemental Table 5). We evaluated 3 types of models: (a) simple exponential decay, (b) segmented decays with an exponential decay until a certain time *t_e_* followed by a different exponential decay or increase after that time, and (c) biexponential models in which the second rate could be decay or an increase (Supplemental Figure 5). Model selection criteria (Bayesian information criterion corrected) indicated that segmented exponential decay models were preferred (Supplemental Table 5). For example, a model incorporating the prior findings of an initial *t*_1/2_ of 44 months (29, 30) and an inflection at 7 years (35) provided a good fit for the data and revealed that after 7 years, there was a slow increase in reservoir size with a doubling time of 23 years (Figure 3B and Supplemental Table 5). Unconstrained models showed an inflection at 3 years followed by a slow increase (Supplemental Table 5). All the biphasic models showed an inflection and subsequent switch to a very slow increase in reservoir size. However, considering the 95% confidence interval, these models are also consistent with no decay in the second phase (*t*_1/2_ = ∞; Supplemental Table 5). Of 16 participants who had longitudinal QVOA measurements, 8 had an increase and 8 had a decrease (Figure 3B).

To provide additional evidence that the reservoir does not continue to decay with long-term ART, we used IPDA measurements to plot the frequency of intact proviruses in resting CD4^+^ T cells as a function of time on suppressive ART (Figure 3C). Data from the previous IPDA decay study (35), which included time points during earlier years of ART, were also plotted and included in our analysis. As with the QVOA results, most IPDA values in later years were higher than expected for decay at *t*_1/2_ = 44.2 months. As for the QVOA data, the IPDA data were not well fit with a single exponential decay model. The best fits were obtained with segmented exponential decay models, including a model with an initial decay (*t*_1/2_ = 46 months) and then an inflection at 7 years, similar to that reported by Peluso et al. (35) (Supplemental Table 6). Subsequently, intact proviruses decayed more slowly, with *t*_1/2_ = 9 years. For IPDA measurements, there was statistical support for slow long-term decay, as the 95% confidence intervals did not include a decay rate of 0. In comparison with the intact proviruses, we found much slower decay for defective proviruses over time (Supplemental Table 7), consistent with other studies (35, 36, 39). Overall, measurements of the latent reservoir in resting CD4^+^ T cells by both the QVOA and the IPDA show that the reservoir decay evident during the first several years of ART does not continue at the same rate.

### Proviral inducibility does not decrease substantially in PWH on very long-term ART.

**Figure 4:**
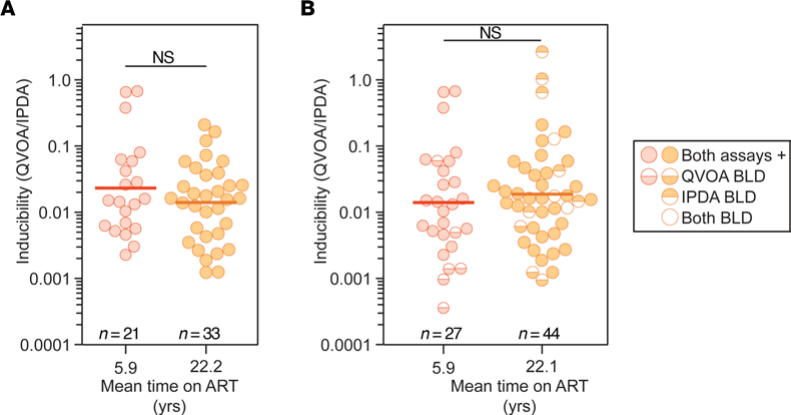
Inducibility of the latent reservoir in PWH on short- and long-term ART. (**A**) QVOA/IPDA analysis of inducibility. Inducibility was measured as the ratio of the frequency of inducible, replication-competent proviruses per 10^6^ resting CD4^+^ T cells over the frequency of intact proviruses per 10^6^ resting CD4^+^ T cells in the same sample (QVOA/IPDA). Short-term ART data are from a study by Bruner et al. (34). The mean duration of treatment with suppressive ART at the time of sampling is shown for each group on the *x* axis. The geometric mean values (thick line) and statistical significance (Student’s *t* test on log-transformed values) are indicated. This analysis was performed using data from samples for which both assays were positive. (**B**) Data from **A** together with results from samples for which one of the frequencies was below the limit of detection (BLD). For these samples, frequencies were estimated as described in the legend to Figure 2.

In light of recent studies of HIV-1 integration sites demonstrating selection for intact proviruses integrated into chromosomal locations associated with reduced gene expression (68), we examined the inducibility of intact proviruses in PWH on very long-term ART. Proviral inducibility has been measured as the ratio between the frequency of cells induced to produce replication-competent virus in the QVOA and the frequency of cells with intact proviruses as measured using IPDA analysis (34, 74). Consistent with previous studies (34, 74), inducibility was less than 10% (Figure 4A). We also plotted the QVOA/IPDA ratios from a previous study in which both assays were done on cells from PWH on ART for a mean of 5.9 years (34). Proviral inducibility in PWH who had been on ART for a mean of 22.4 years was not significantly different from that previously observed in PWH for a mean of only 5.9 years. For this analysis, we used data from samples for which both assays were positive. However, in samples from some donors, high frequencies of inducible, replication-competent proviruses were detected by QVOA despite intact proviral frequencies below the limit of detection of the IPDA (Figure 4B and Supplemental Figure 4). This result indicates high proviral inducibility in some samples. When those higher ratios are included in the analysis, there is a non-significant trend toward higher inducibility for the long-term cohort. The proliferation of rare cells with highly inducible proviruses may explain why QVOA values tended to increase over the long run while the total population of proviruses remained relatively stable or decreased slightly.

### Large clones of inducible, replication-competent proviruses contribute to reservoir persistence in PWH on very long-term ART.

**Figure 5:**
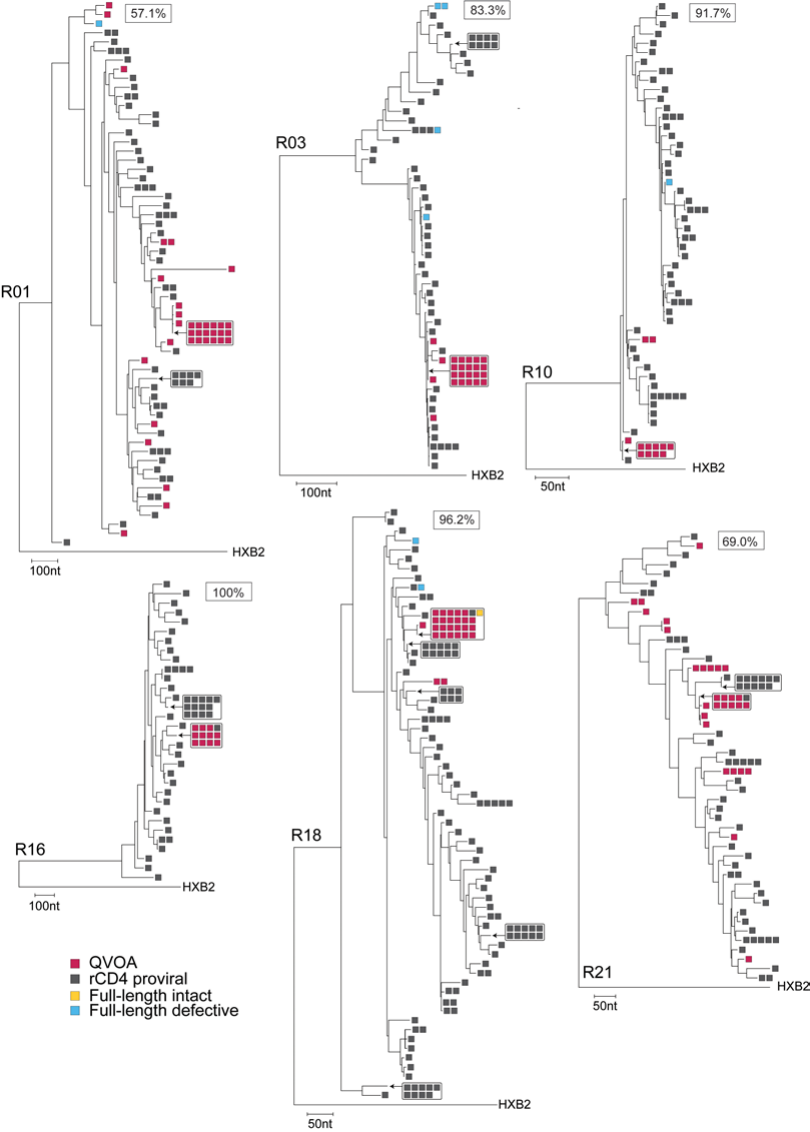
Large expanded CD4^+^ T cell clones dominate the latent reservoir of PWH on very long-term ART. Maximum-likelihood phylogenetic trees of *env* sequences for 6 representative participants (R01, R03, R10, R16, R18, and R21), each rooted using HXB2, are shown. Single-genome sequencing of *env* was performed on cDNA reverse-transcribed from viral RNA extracted from supernatants of QVOA wells scored positive for viral outgrowth (red) or on proviral DNA from resting CD4^+^ T cells (rCD4, gray). Near-full-length genome sequences were obtained using the same proviral DNA and are annotated as intact (yellow) or defective (blue). Genetic distance is represented by the scale in nucleotides. A measure of the clonality of QVOA isolates (see article text) is indicated as a boxed value for each participant.

**Figure 6:**
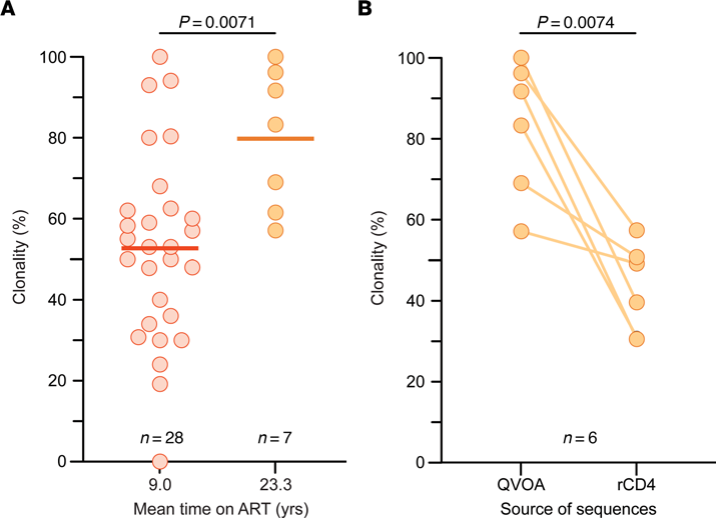
Clonality of the latent reservoir of PWH on very long-term ART. (**A**) Clonality of QVOA isolates. Clonality was estimated as the fraction of *env* sequences that exactly matched another *env* sequence from an independent isolate obtained in the same QVOA. Clonality of outgrowth sequences for participants with over 10 positive QVOA outgrowth wells was compared between PWH on very long-term ART (R01, R02, R03, R10, R16, R18, and R21; orange symbols) and PWH on ART for an average of 9.0 years (red symbols) from 5 previous studies (45–47, 57, 75). In those 5 studies, clonality measurements included sequences obtained only from the first time point using the same QVOA methods with only 1 round of stimulation and from participants who experienced no analytical treatment interruptions and had no additional treatments besides ART. The mean values (thick line) and statistical significance (Student’s *t* test) are indicated. (**B**) Difference in clonality between QVOA isolates and proviral *env* sequences. Clonality of outgrowth sequences for very long-term ART participants with over 10 positive QVOA outgrowth wells (R01, R03, R10, R16, R18, and R21) was compared with the clonality of proviral sequences obtained from resting CD4^+^ T (rCD4) cells of the same participant. Statistical significance (Student’s paired *t* test) is indicated.

Large clones of infected CD4^+^ T cells arise from the proliferation of infected cells and contribute to viral persistence (40–47). We examined whether the absence of reservoir decay with long-term ART (Figure 3) is a result of infected-cell proliferation. We conducted single-genome *env* sequencing of viral RNA in supernatants of p24^+^ QVOA wells, focusing on 6 representative study participants from whom more than 10 independent viral isolates were obtained (R01, R03, R10, R16, R18, and R21). To evaluate how the inducible, replication-competent proviruses fit into the overall landscape of persistent proviruses, we also did *env* and near-full-length sequencing on proviral DNA from these participants. For all samples analyzed, we observed the expected participant-specific clustering of sequences (Supplemental Figure 6). For all 6 participants, the majority of viruses growing out in the QVOA were identical to isolates from other independent QVOA wells (Figure 5). These replication-competent isolates represented only a small fraction of the total pool of *env*^+^ proviruses, many of which were likely defective outside of the *env* region. To estimate the clonality of inducible, replication-competent proviruses, we used a previously described metric (45–47), the ratio of the number of sequences precisely matching at least 1 other sequence from the same sample to the total number of sequences obtained. The mean clonality of the inducible, replication-competent proviruses was 79.8% (range 57.1%–100%) for 7 participants with more than 10 p24^+^ QVOA wells (Figure 6A). Phylogenetic trees for 6 of these participants are shown in Figure 5. In comparison, the mean clonality of viral isolates from participants in 5 previous studies (45–47, 57, 75) of PWH on ART for an average of 9 years was only 52.7% (Figure 6A). The significant increase (*P* = 0.0071) in clonality for PWH on very long-term ART suggests that infected T cell clones generally increase in size over time on ART.

We also examined clonality in proviral *env* sequences. We obtained an average of 65 proviral sequences per donor and found sets of identical sequences for each donor (Figure 5). The clonality of those proviruses was 41.3%, significantly lower than the clonality of viruses that grew out in the QVOA (*P* = 0.0074; Figure 5 and Figure 6, A and B). The finding that replication-competent proviruses are a part of a more diverse set of proviruses, even after 20 years, demonstrates that the presence of large replication-competent clones was not due to an overall lack of reservoir diversity. Instead, infected cells must have proliferated in vivo. Viral isolates from the QVOA did not match any of the proviral sequences for 3 of the 6 participants (R01, R03, and R10), while only one QVOA sequence matched a proviral sequencing for R16, R18, and R21 (Figure 5). Thus, inducible, replication-competent proviruses detected in outgrowth assays make up a small proportion of the total diversity of the persistent proviral population. To determine whether there were differences in latent reservoir clonality for participants with high versus low levels of viral outgrowth, we also performed proviral sequencing on resting CD4^+^ T cells for 4 participants with 1 or 0 positive wells in the QVOA (R06, R07, R11, and R17). We obtained an average of 50 sequences per donor. The mean clonality of proviruses from resting CD4^+^ T cells of participants with low outgrowth was 51.5%, not significantly different from that of participants with a higher frequency of replication-competent virus (*P* = 0.5090; Supplemental Figure 7).

The large number of proviruses identified in proviral *env* sequencing but not captured by QVOA could be explained by the presence of proviruses with defects elsewhere in the genome and/or by the presence of viruses that are intact but not induced by a single round of T cell activation. Therefore, we performed near-full-length proviral sequencing for 9 participants, including those with high and low viral outgrowth (R01, R03, R06, R07, R10, R11, R16, R17, and R18). Of a total of 195 sequences, 25 had an intact *env* gene and were included in phylogenetic trees (Figure 5 and Supplemental Figure 7). However, some of these proviruses had defects elsewhere in the genome, and overall, we observed a very high proportion of defective sequences. Only 1 of the near full-length proviral sequences was fully intact (Figure 5 and Supplemental Figure 8). It matched the large set of 23 inducible, replication-competent proviruses detected in participant R18 (Figure 5). Many of the remaining sequences matched previously identified proviral sequences from resting CD4^+^ T cells but were defective outside of *env*. Interestingly, the largest clones identified by proviral *env* sequencing in participants with little or no viral outgrowth (R06 and R11) matched defective proviruses identified through near-full-length proviral sequencing. The nature of the defects is described in detail in Supplemental Figure 8 and is similar to previous reports (33, 34, 38, 76). The finding that outgrowth sequences rarely match near-full-length genome sequences stems from the large diversity of proviruses that persist in treated PWH and the large fraction of these that are defective, as observed in our study (Figure 2D) and other studies (33, 71). Thus, capturing the full genomic sequence (and the integration sites) of the expanded clones that give rise to outgrowth in the QVOA will likely require the development of novel full-length sequencing assays that have a much higher throughput than current assays. Much of the proviral diversity we captured by *env* proviral sequencing is likely attributable to defective proviruses, but some is due to a population of intact, non-induced proviruses.

Through sequencing of individual QVOA wells positive for viral outgrowth and single proviruses from resting CD4^+^ T cells, we have independently collected multiple viral variants that are the same and that may have originated from a single infected cell in vivo. Since we are sampling a small proportion of each participant’s reservoir, the observed presence of large sets of identical sequences suggests that some infected cells have proliferated extensively, which may explain why the reservoir does not continue to decay and appears to be slightly larger in PWH on very long-term ART.

## Discussion

The frequency and decay rate of inducible, replication-competent proviruses have not been extensively analyzed in PWH on very long-term suppressive ART. Here, we used the QVOA to demonstrate that inducible, replication-competent proviruses are readily detectable in PWH on suppressive ART for a mean of 22 years and do not continue to decay with the 44.2-month half-life defined in short-term decay studies (29, 30). Instead, after an initial period of rapid decay of labile populations of infected cells defined in recent studies (77, 78), the reservoir decays with a half-life of 44.2 months for the first several years of ART. Then, there is an inflection after which the frequency of latently infected cells, as measured by the QVOA, slowly increases with a doubling time of 23 years, although the 95% confidence intervals are wide and include the possibility of a zero rate of increase or even a very slow decay (Figure 3B and Supplemental Table 5). Further studies in other populations of PWH on very long-term ART will be needed to determine whether the reservoir is actually increasing or simply not decaying as rapidly as expected based on the first several years of ART. The QVOA results were supported by an additional assay, the IPDA, which also showed that intact proviruses do not continue to decay at the rates observed during the first years of ART; intact proviruses decayed with a half-life of 46 months for the first 7 years of ART and then with a longer half-life of 9 years (Figure 3C and Supplemental Table 6). Overall, measurements of the latent reservoir in resting CD4^+^ T cells by both the QVOA and the IPDA show that the reservoir decay evident during the first several years of ART does not continue at the same rate. Replication-competent viral isolates from PWH on long-term ART often had *env* sequences identical to those of other independent isolates from the same blood sample (Figure 5), consistent with clonal expansion as a major mechanism of reservoir persistence (42–48, 50–52). Together, these results indicate that the slow decay in the frequency of inducible, replication-competent proviruses observed in the first several years of ART is counteracted by proliferation such that there is no net decay over the long term. Given this, continued ART is necessary regardless of time on treatment.

Important recent studies of HIV-1 integration sites have suggested that with long-term ART there is a selection for proviruses integrated in less transcriptionally active regions of the genome with less potential to cause viral rebound in the case of ART interruption (66–68). Although these studies did not directly demonstrate that proviruses with integration sites located in such genomic regions are non-inducible, the findings suggest that inducible, replication-competent proviruses might be infrequent or absent in PWH on very long-term ART and that the inducibility of intact proviruses would decrease over time on ART. However, we detected inducible, replication-competent viruses in the majority of our participants (36/42, 86%) using the QVOA (Figure 2, A and B, and Figure 3, A and B). In fact, the frequency of cells with inducible, replication-competent virus appears to be slightly higher in PWH on long-term ART because of infected-cell proliferation. We also found no significant change in the proviral inducibility over very long-term ART (Figure 4). Samples with no outgrowth generally had low levels of intact proviruses by the IPDA, suggesting that the absence of outgrowth was due to low reservoir size rather than reduced inducibility. Therefore, recently described changes in the proviral landscape do not appear to affect overall inducibility of latent proviruses as measured in the QVOA. The finding that intact proviruses in PWH on long-term ART are mainly integrated within transcriptionally inactive, non-genic regions of the genome (68) may be reconciled with our results if some of those proviruses can be induced. It is also possible that the outgrowth that we readily detect in the QVOA is coming from minor variants with integration sites in euchromatin. In any event, our study clearly shows that inducible, replication-competent proviruses are abundant in PWH on long-term ART and may cause rebound if ART is stopped. Identification of integration sites of inducible, replication-competent proviruses in PWH on very long-term ART will clarify this issue.

Our findings indicate that reservoir decay slows and may even be reversed in PWH on long-term ART. To determine whether this change reflects the proliferation of infected cells, we sequenced replication-competent viral isolates and proviral DNA from resting CD4^+^ T cells of study participants. For 6 participants from whom we obtained 10 or more independent isolates of replication-competent virus, we found that 79.8% of isolates had *env* sequences identical to those of other isolates from the same blood sample despite high sequence diversity evident in proviral *env* sequencing (Figure 5). Although clonality cannot be definitively established without integration site analysis, our results are consistent with the idea that the proliferation of infected cells is a primary factor in reservoir stability (42–48, 50–52). Although longitudinal tracking of the clonality of inducible, replication-competent proviruses over a 20-year interval is difficult to achieve because of limited sample availability, we compared our results with similar QVOA studies in PWH on ART for shorter periods of time. We found that the level of clonality for PWH on very long-term ART (average 23.3 years) was significantly greater than the clonality observed in PWH on ART for an average of only 9 years (45–47, 57, 75) (Figure 6). The increase in clonality is consistent with near-full-genome sequencing results of Lian et al., who found large clones of intact proviruses in PWH on very long-term ART (68), and with previous studies indicating an increase in reservoir clonality over time on ART (44, 79–82) and the long-term stability of infected CD4^+^ T cell clones compared with uninfected clones (83). The proliferation of certain infected cell clones, perhaps a rare population with higher inducibility, may explain the slowing and possible reversal of net reservoir decay. Other factors, including an age-related decline in immune competence, may also contribute to the change in reservoir decay rate.

We chose the QVOA as the primary assay for reservoir size because it quantitates proviruses that give rise to exponential viral outgrowth, reflecting the potential for viral rebound (31, 32). Importantly, this method provides a measure of inducibility, a critical factor in determining whether genetically intact proviruses can contribute to rebound. Inducibility is not addressed by the IPDA or other PCR-based methods. Importantly, the QVOA is not skewed by the large proportion of defective proviruses that persist in treated individuals (33). Thus, the QVOA remains the gold standard method to measure the frequency of cells with replication-competent and inducible proviruses that are the barrier to cure (6, 31, 32).

Our study has limitations. QVOA results from the early years of ART were only available for 4 of 42 study participants. Thus, for the most part we are comparing assay results from 2 different groups of PWH. Nevertheless, the lack of any significant difference in QVOA values between the 2 groups strongly supports our conclusion that reservoir decay does not continue. Most participants were older (mean age 40.8) and had low CD4 nadirs (mean 161 cells/μL). It is possible that immune recovery, age, and age-related declines in immune competence could contribute to the reservoir dynamics observed here. In future studies, it will be important to examine reservoir decay in PWH who start ART at a younger age with a higher CD4 nadir. It has been argued that stimulation of CD4^+^ T cells with phytohemagglutinin (PHA) and IL-2 in the QVOA may induce proviruses that would not be induced in vivo. However, PHA has been used for decades as a polyclonal mimic for antigen stimulation (84–86), and we have demonstrated antigen-mediated induction of viral gene expression in a study participant who was on long-term ART and had a strong CD4^+^ T cell response to CMV. Using autologous dendritic cells pulsed with CMV antigens, we could readily induce expression of HIV-1 RNA from an infected CD4^+^ T cell clone from this participant (M. Moskovljevic, unpublished observations). Thus, we believe that at least some of the replication-competent proviruses induced in the QVOA are also capable of producing virus following in vivo stimulation with the relevant antigen. Analytical treatment interruption studies would be more revealing but difficult to justify. It is important to note that the QVOA may underestimate the size of the latent reservoir because it does not capture all the intact proviruses that are potentially inducible after multiple rounds of T cell activation (33, 47). The IPDA, on the other hand, captures all genetically intact proviruses but may overestimate the frequency of cells harboring intact proviruses because some defective proviruses have small deletions not encompassed by the 2 IPDA amplicons (34). Additionally, many replication-competent proviruses are not induced by a single round of T cell activation in the QVOA (33, 47), which explains why IPDA values are generally much higher than QVOA values. Overall, the QVOA and IPDA provide definitive minimal and maximal estimates, respectively, of latent reservoir size. In our study, both assays showed that reservoir decay does not continue at the previously predicted rate in PWH on long-term ART, strengthening the conclusions.

Our analysis of reservoir decay in PWH on very long-term ART has shown that after initial decay for several years there is no further decrease. There may be a slow increase in the size of the inducible, replication-competent reservoir due to infected-cell proliferation. Our findings clearly demonstrate that the reservoir is maintained over 2 decades of treatment and is still a major barrier to HIV-1 cure.

## Methods

### Study participants.

Participants were selected based on a documented history of suppression of viremia on ART for 14–27 years with generally undetectable plasma HIV-1 RNA levels and no lapses in treatment. Blips, isolated single measurements above the limit of detection, were not considered treatment failure. We received deidentified leukapheresis samples from 8 participants in the UCSF SCOPE cohort, 18 participants at the University of Toronto, and 1 participant at the University of Pennsylvania. We also recruited 15 participants from the Johns Hopkins Hospital Bartlett Clinic who provided peripheral blood samples. Participant characteristics are shown in Supplemental Table 1. Graphical depictions of participant viral loads and CD4 counts are shown in Figure 1 and Supplemental Figures 1–3.

### Resting CD4^+^ T cell isolation.

Peripheral blood mononuclear cells (PBMCs) were isolated from blood and leukapheresis samples using Ficoll-Hypaque gradients. CD4^+^ T cells were isolated by negative selection using the EasySep Human CD4^+^ T Cell Enrichment Kit (STEMCELL Technologies). Resting CD4^+^ T cells were isolated from the CD4^+^ T cell preparations by negative selection of cells expressing CD25, CD69, and HLA-DR (CD25 MicroBeads II, 130-092-983; CD69 MicroBead Kit II, 130-092-355; anti–HLA-DR MicroBeads, 130-046-101; Miltenyi Biotec). For some participants, PBMCs were viably frozen and thawed before CD4^+^ T isolation.

### Quantitative viral outgrowth assay (QVOA).

Purified resting CD4^+^ T cells were plated in five 5-fold serial dilutions as previously described (31, 37). To induce virus production, the cells were activated with 0.5 μg/mL PHA and irradiated PBMCs from healthy donors. The next day, PHA was removed, and the HIV-permissive cell line MOLT-4/CCR5 (American Type Culture Collection; ref. 87) was added to amplify virus released from CD4^+^ T cells. Cells were cultured in medium containing 2% T cell growth factor supernatant (cytokine-rich supernatant prepared as previously described; refs. 31, 32) and 100 U/mL recombinant human IL-2 for 21 days with regular medium replacement. On days 14 and 21, an HIV-1 p24 capsid antigen ELISA (NEK050B001KT, PerkinElmer) was used to detect HIV-1 in culture supernatant. The results were expressed as infectious units per million (IUPM) resting CD4^+^ T cells on day 21, calculated using maximal-likelihood limiting-dilution statistics with IUPMStats v1.0, as described by Rosenbloom et al. (88). For assays in which no outgrowth was observed, a posterior median estimate is given. This value reports the median of the Bayesian posterior distribution, using a uniform prior (88).

### Intact proviral DNA assay (IPDA).

To measure the levels of genetically intact, 3′ defective/hypermutated, and 5′ defective proviruses, genomic DNA was extracted (QIAamp DNA Mini Kit 51306, Qiagen) from each participant’s resting CD4^+^ T cells and used in the IPDA as previously described (34).

### Viral RNA isolation and cDNA synthesis.

Viral RNA from centrifuged (5,000*g* at 4°C for 15 minutes) supernatants of p24^+^ QVOA wells was isolated using a 96-well spin plate according to the manufacturer protocol (R1041, Zymo Research). The eluted RNA was subjected to *env*-specific complementary DNA (cDNA) synthesis. In a 96-well PCR plate, the cDNA was made by mixing of 20 μL of the RNA isolate, 2.5 μL of 10 mM dNTPs, and 2.5 μL of 2 μM envelope-specific primer env3out (5′-TTGCTACTTGTGATTGCTCCATGT-3′) (89). The RNA was denatured at 65°C for 10 minutes and placed on ice. After 1 minute, 10 μL of 5× First-Strand Buffer, 0.5 μL of 0.1 M DTT, 13.5 μL of sterile free water, 0.5 μL of 40 U/μL RNaseOUT recombinant ribonuclease inhibitor, and 0.5 μL of 200 U/μL SuperScript III Reverse Transcriptase were added (18080044, 10777019, Invitrogen). The samples were then incubated at 50°C for 50 minutes and then inactivated at 85°C for 10 minutes.

### Sequencing of viral isolates and proviruses in resting CD4^+^ T cells.

For sequencing of outgrowth virus, full-length *env* was amplified using the cDNA from each p24^+^ QVOA well in nested outer and inner PCRs. For proviral sequencing, full-length *env* was amplified from extracted genomic DNA from resting CD4^+^ T cells (QIAamp DNA Mini Kit 51306, Qiagen) using the same nested outer and inner PCRs. The outer and inner PCR reactions were performed as previously described (89) with a change to the primer env3in (5′-TTTGACCACTTGCCACCCAT-3′). The PCR products were run on 1% agarose. All PCR products from plates that were at limiting dilution (<30% positive wells across 12 replicates) were analyzed by Sanger sequencing (Genewiz/Azenta) using 4 previously described primers (59). Near-full-length proviral sequencing was done by AccelevirDx. High–molecular weight genomic DNA was isolated from between 2 million and 5 million CD4^+^ T cells (Gentra Puregene Kit 158043, Qiagen). Near-full-length amplification of single templates was performed at limiting dilution using nested PCR, and amplicons from plates with less than 30% positive reactions were characterized via rapid gel electrophoresis and sequenced. We performed library preparation (plexWell 384 [PW384], seqWell) and short-read 2 × 300 bp sequencing (MiSeq Reagent Kit v3, MS-102-3003, Illumina) on clonal provirus amplicons, according to the manufacturer’s instructions. A consensus sequence was produced for each amplicon using an in-house short-read next-generation sequencing analysis pipeline, which performs quality trimming, adapter and contaminant removal, and iterative assembly methods to produce single contigs for each provirus amplicon. Resulting contigs were screened for depth of read coverage and consistency with expected size, and those passing quality control metrics were subject to further analysis. Correct assembly was further confirmed by global alignment with iterative refinement, including the HIV-1 sequence HXB2 (GenBank: K03455) for reference and base position coordinates.

### Sequence alignment and phylogenetic analysis.

After Sanger or next-generation sequencing, contigs were checked for quality and assembled in Geneious. In BioEdit (: https://bioedit.software.informer.com/download/), sequences were aligned to HXB2 and hypermutants were removed. Unique sequences were identified with ElimDupes (https://www.hiv.lanl.gov/content/sequence/elimdupesv2/elimdupes.html) and used to generate maximum-likelihood phylogenetic trees with PHYML as previously described (59) and with bootstrapping of 100 replicates. Trees were visualized in MEGA11 (https://www.megasoftware.net/), and annotations were made in Adobe Illustrator.

To demonstrate distinct HIV-1 variants in each participant, unique outgrowth and proviral sequences from all participants were aligned to HXB2 using HIVAlign by MAFFT (Los Alamos National Laboratories, https://www.hiv.lanl.gov/content/sequence/VIRALIGN/viralign.html). The resulting alignment was used to build a neighbor-joining tree with Treemaker from Los Alamos National Laboratories (https://www.hiv.lanl.gov/content/index) under a general time reversible (GTR) distance model.

### Modeling of decay processes.

We used a mixed-effects modeling approach to fit different models to the long-term decay of infected cells assayed by QVOA or IPDA. We tried the following models for both the QVOA and IPDA data sets: (a) simple exponential decay; (b) segmented exponential, that is, an exponential decay with rate *r*_1_ until a certain time *t_e_* followed by an exponential decay/increase with different rate *r*_2_ after that time; and (c) biexponential, where the second rate could be decay or increase (Supplemental Figure 5). The difference between (b) and (c) is that (b) assumes that the level of infected cells is changing with one rate which switches at time *t_e_*, whereas (c) assumes that the level of infected cells is changing as a sum of 2 exponentials with different rates throughout the period of observation. Biologically, one could say that (b) assumes a mean rate of change of the infected cell population which switches after some time (perhaps because of biological changes in the system such as increased proliferation), whereas (c) assumes that there are 2 (effective) populations whose levels change with different rates throughout. Clearly, there could be more populations with different rates of change, but the data do not support statistically estimating more than 2 rates.

Previous studies (29, 30) with QVOA data showed that over the first 7 years after ART initiation, an exponential decline with half-life *t*_1/2_ = 44 months described the data well. This was the reason to try the single exponential decay over the current longer period of observation (of up to 27 years). This is also why we tested segmented fits with the first phase half-life fixed at 44 months and breakpoint at 7 years. The best fit results for each type of model (single exponential, segmented exponential, or biexponential) are shown in Supplemental Table 5 for the QVOA, Supplemental Table 6 for the IPDA, and Supplemental Table 7 for defective proviruses.

### Statistics.

Statistical significance was calculated with the 2-tailed Student’s *t* test and Spearman’s rank correlation using GraphPad Prism 9.0. A *P* value of 0.05 was considered significant.

### Study approval.

Sample collection and processing were performed according to protocols approved by the University of Pennsylvania (Philadelphia FIGHT), UCSF (SCOPE, Human Research Protection Program), Johns Hopkins University Bartlett Clinic, and University of Toronto (Unity Health Toronto) Institutional Review Boards. All participants provided written informed consent.

### Data availability.

All HIV-1 sequences are available in the NCBI’s GenBank (accession numbers OQ820255–OQ820694). Data for figures can be found in the Supporting Data Values file.

## Author contributions

NFM, JV, EJF, JL, MM, MAG, BML, VH, and KR performed experiments. AS assembled participant clinical histories. CZ, RMR, and ASP performed mathematical modeling of decay processes. HM and GML conducted near-full-length sequencing. KL, PT, KM, LJM, EB, CK, RH, and SGD provided participant samples. NFM, JV, FRS, RFS, and JMS investigated the results. NFM, RFS, and JMS wrote the manuscript with feedback from all authors.

## Supplementary Material

Supplemental data

Supporting data values

## Version 1 07/18/2023

In-Press Preview

## Version 2 09/01/2023

Electronic publication
