# Supplementary material for: The latent reservoir of inducible, infectious HIV-1 does not decrease despite decades of antiretroviral therapy
Source: J Clin Invest. 2023 Sep 1;133(17):e171554. doi: 10.1172/JCI171554 (PMC10471168; doi:10.1172/JCI171554)
Supplement: Supplemental data [file jci-133-171554-s007.pdf]

Supplementary Information for:

The latent reservoir of inducible, infectious HIV-1 does not decrease despite decades of antiretroviral therapy

Natalie F. McMyn[1,] Joseph Varriale[1], Emily J. Fray[1], Carolin Zitzmann[2], Hannah MacLeod[3], Jun Lai[1], Anushka Singhal[1], Milica Moskovljevic[1], Mauro A. Garcia[1], Brianna M. Lopez[1], Vivek Hariharan[1], Kyle Rhodehouse[1], Kenneth Lynn[4,5], Pablo Tebas[5], Karam Mounzer[6], Luis J. Montaner[4], Erika Benko[7], Colin Kovacs[7], Rebecca Hoh[8], Francesco R. Simonetti[1], Gregory M. Laird[3], Steven G. Deeks[8], Ruy M. Ribeiro[2], Alan S. Perelson[2], Robert F. Siliciano[1,9], and Janet M. Siliciano[1,*]

Figures

Figure S1. Plasma HIV-1 RNA levels and CD4$^+$ T counts of participants R1-R15
Figure S2. Plasma HIV-1 RNA levels and CD4$^+$ T counts of participants R16-30
Figure S3. Plasma HIV-1 RNA levels and CD4$^+$ T counts of participants R31-R42
Figure S4. Correlation between reservoir assays
Figure S5**.** Fits to the QVOA data using a linear, segmented exponential and biexponential models
Figure S6. Participant-specific clustering of *env* sequences
Figure S7. Large expanded clones dominate the population of persistent proviruses
Figure S8. Near full-length sequences of proviruses from PWH on very long-term ART

Tables

Table S1. Characteristics of study participants
Table S2. Changes in drug regimens during suppressive ART
Table S3. Blips during suppressive ART
Table S4. Decay of cells with inducible, replication-competent proviruses as measured by QVOA
Table S5. Models for decay of inducible, replication-competent HIV-1 with very long-term ART
Table S6. Best fit models for decay of intact proviruses as measured by IPDA
Table S7. Best fit models for decay of defective proviruses as measured by IPDA

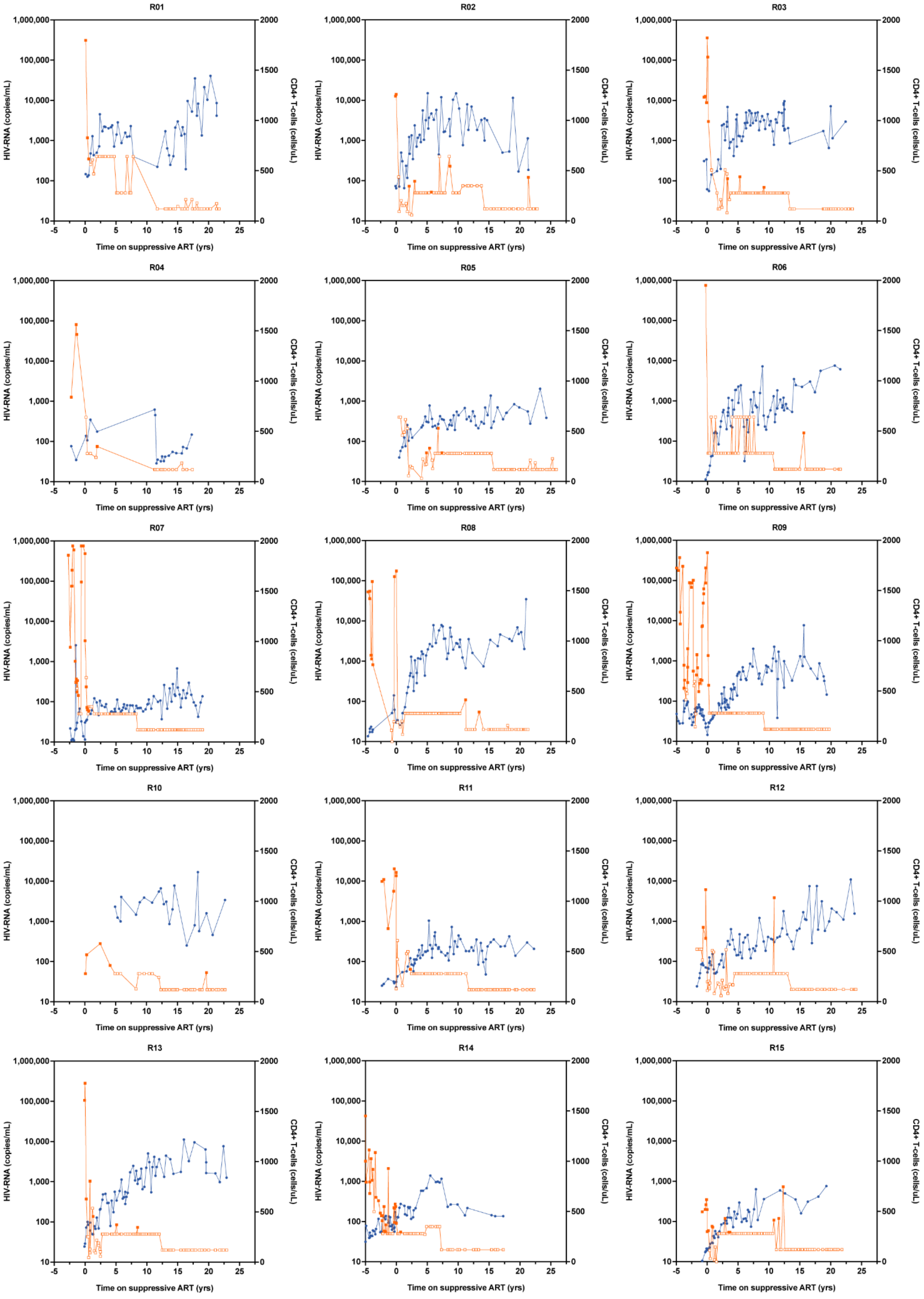
R01
R02
R03
R04
R05
R06
R07
R08
R09
R10
R11
R12
R13
R14
R15
HIV-RNA (copies/mL)
10
100
1,000
10,000
100,000
1,000,000
CD4+ T-cells (cells/uL)
0
500
1000
1500
2000
Time on suppressive ART (yrs)
-5
0
5
10
15
20
25

Figure S1. Plasma HIV-1 RNA levels and $CD4^+$ T counts of participants R01-R15. Levels of plasma HIV-1 RNA in copies/ml (solid orange squares) and $CD4^+$ T cells in cells/μl (blue circles) are plotted as a function of time on suppressive ART. Plasma HIV-1 RNA values below the limit of detection are denoted using open orange squares and plotted at the limit of detection of the assay used. Fluctuations in plasma HIV-1 RNA levels prior to initiation of suppressive ART mostly reflect regimen changes. Fluctuations after initiation of suppressive ART largely reflect differences in the limit of detection of the assay used. The limit of detection for most assays was 50 copies/ml or 20 copies/ml. ART regimens for each participant are listed in **Table S2**.

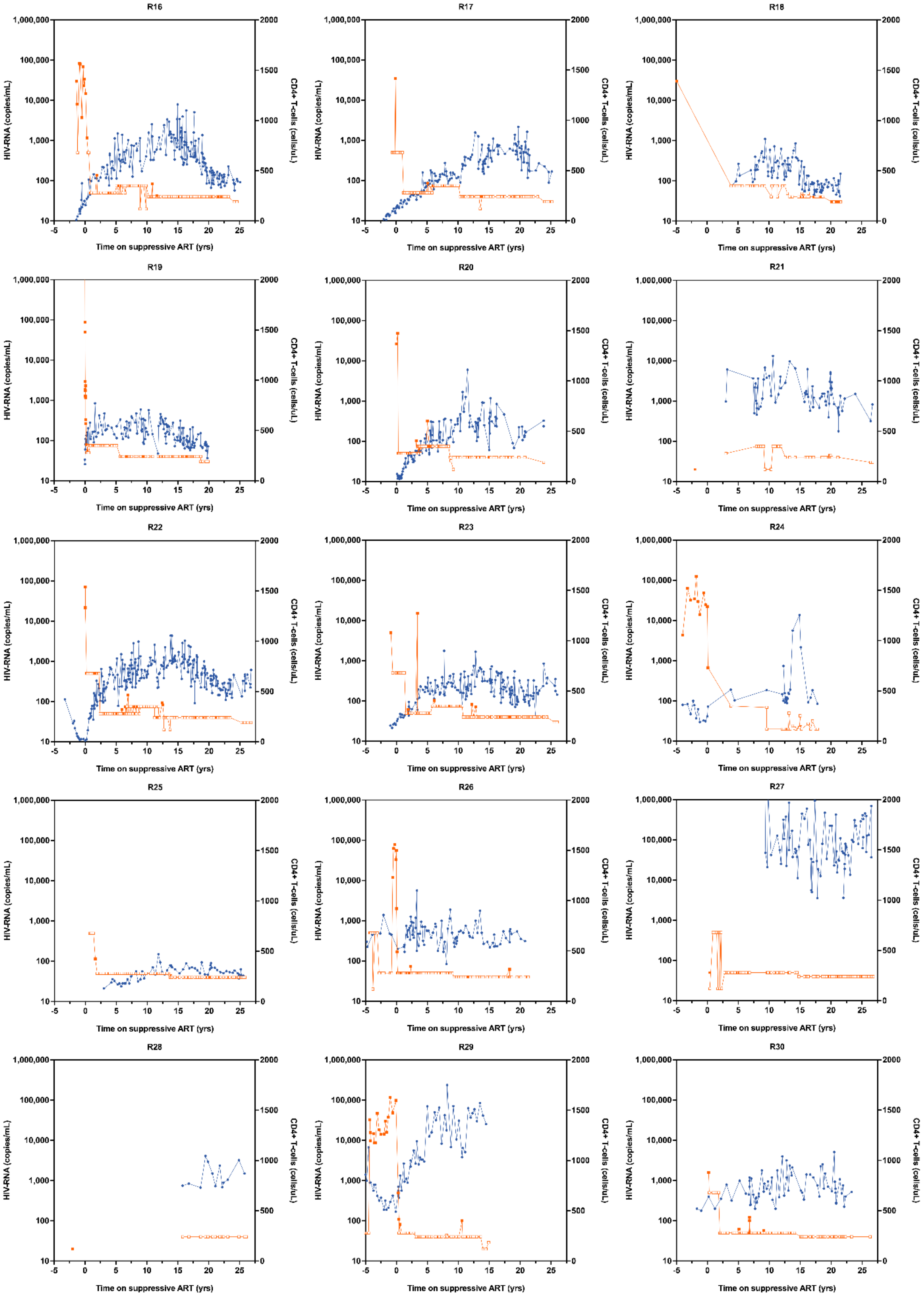

R16
R17
R18
R19
R20
R21
R22
R23
R24
R25
R26
R27
R28
R29
R30
HIV-RNA (copies/mL)
10
100
1,000
10,000
100,000
1,000,000
CD4+ T-cells (cells/uL)
0
500
1000
1500
2000
Time on suppressive ART (yrs)
-5
0
5
10
15
20
25

Figure S2. Plasma HIV-1 RNA levels and $CD4^+$ T counts of participants R16-R30. Levels of plasma HIV-1 RNA in copies/ml (solid orange squares) and $CD4^+$ T cells in cells/µl (blue circles) are plotted as a function of time on suppressive ART. Plasma HIV-1 RNA values below the limit of detection are denoted using open orange squares and plotted at the limit of detection of the assay used. Fluctuations in plasma HIV-1 RNA levels prior to initiation of suppressive ART mostly reflect regimen changes. Fluctuations after initiation of suppressive ART largely reflect differences in the limit of detection of the assay used. The limit of detection for most assays was 50 copies/ml or 20 copies/ml. ART regimens for each participant are listed in **Table S2**.

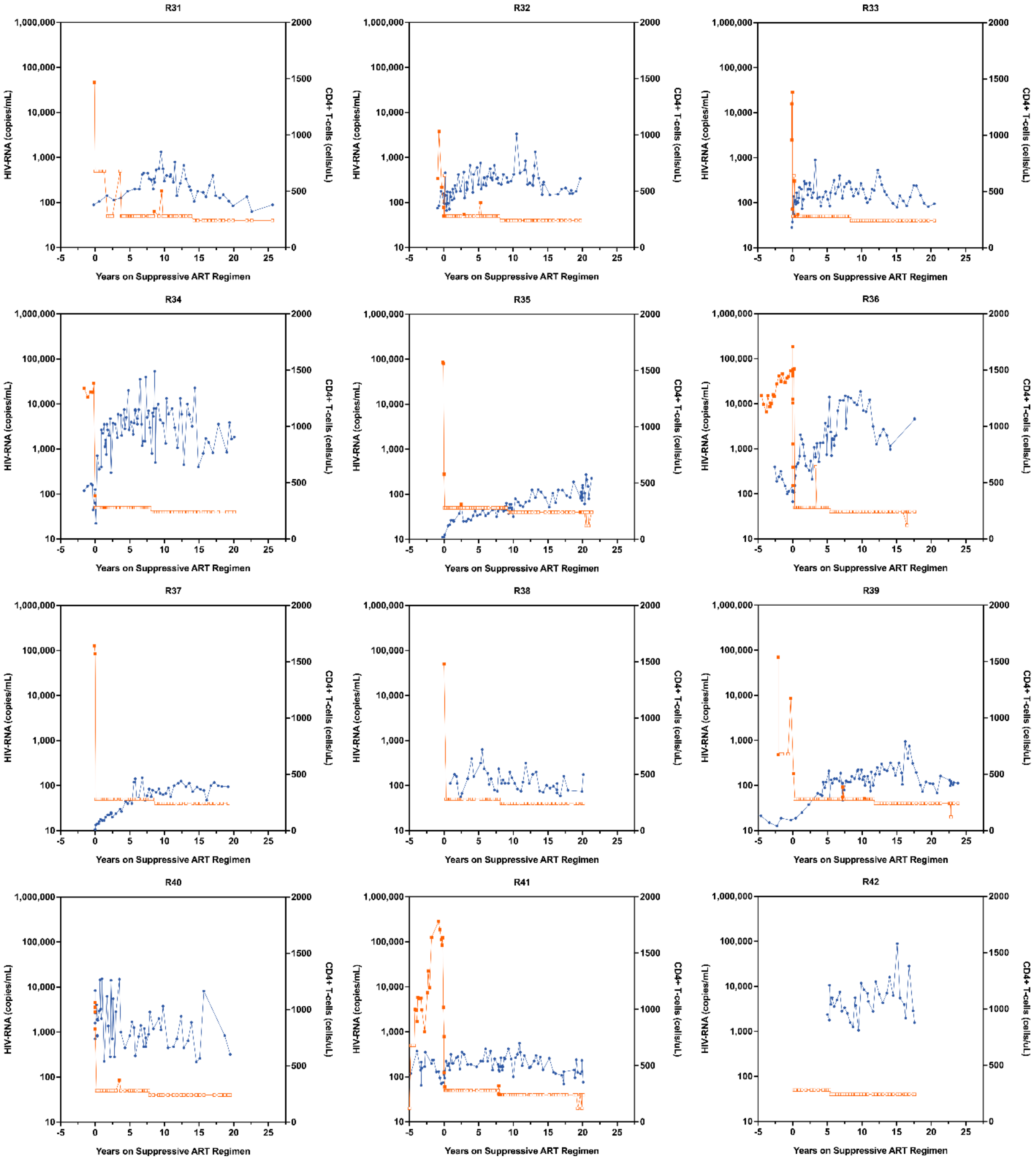

Figure S3. Plasma HIV-1 RNA levels and CD4$^+$ T counts of participants R31-R42. Levels of plasma HIV-1 RNA in copies/ml (solid orange squares) and CD4$^+$ T cells in cells/μl (blue circles) are plotted as a function of time on suppressive ART. Plasma HIV-1 RNA values below the limit of detection are denoted using open orange squares and plotted at the limit of detection of the assay used. Fluctuations in plasma HIV-1 RNA levels prior to initiation of suppressive ART mostly reflect regimen changes. Fluctuations after initiation of suppressive ART largely reflect differences in the limit of detection of the assay used. The limit of detection for most assays was 50 copies/ml or 20 copies/ml. ART regimens for each participant are listed in **Table S2.**

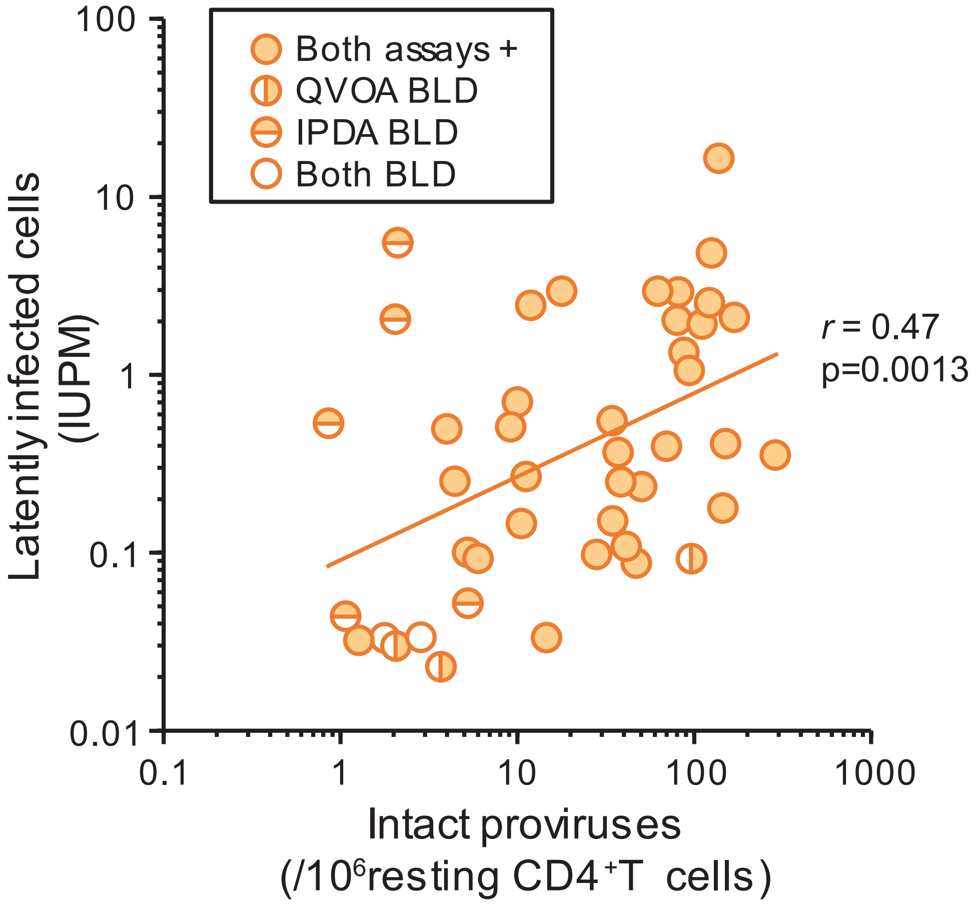

Figure S4. Correlation between reservoir assays. For 44 samples from PWH on very long-term ART, enough cells were available to perform both the QVOA and IPDA. The mean duration of suppressive ART at the time of sampling for these samples was 22.1 years. Samples for which infected cell or proviral frequencies were below the limit of detection (BLD) of the relevant assays are indicated by unshaded half or full circles. In these cases, frequencies were estimated as described in **Figure 2**. Correlation between assay values was evaluated using the Spearman rank correlation.

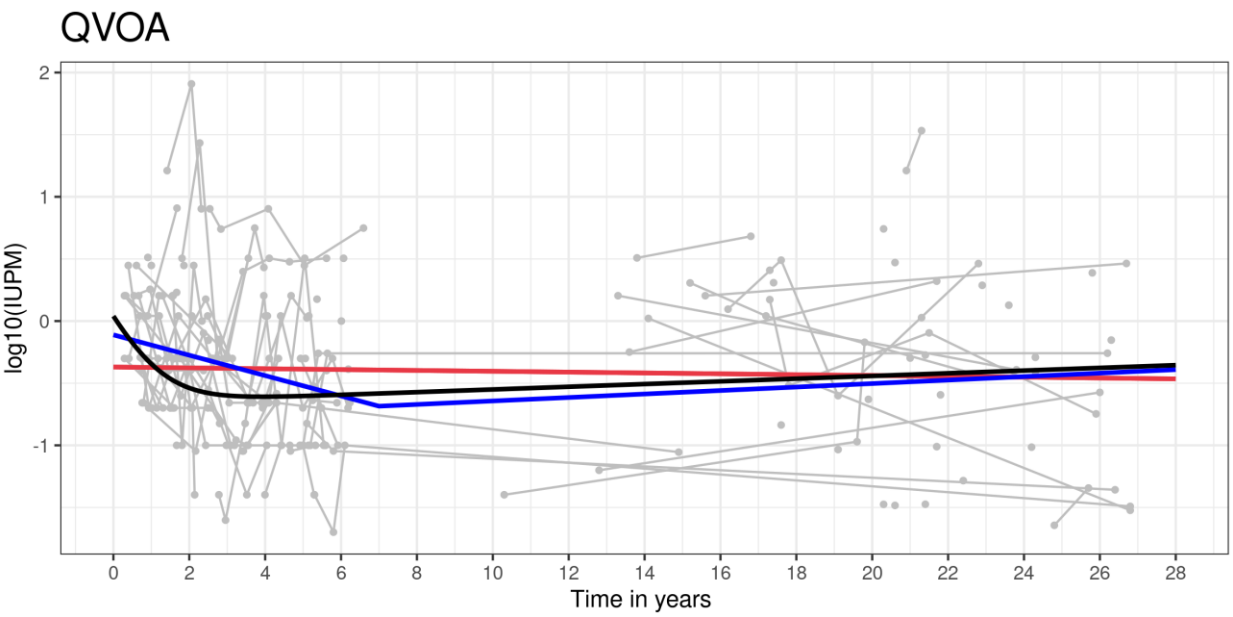

Figure S5. Fits to the QVOA data using a linear model (red), segmented exponential model (blue) and a biexponential model (black). For the linear model, the half-life is 89 yrs. For the segmented model, the first phase half-life was fixed at 44 months, the break point was fixed at 7 years, and the estimated second phase doubling time was 23 years. For the biexponential model, the estimated first phase half-life was 6 months and the second phase doubling time was 28 years (**Table S5**).

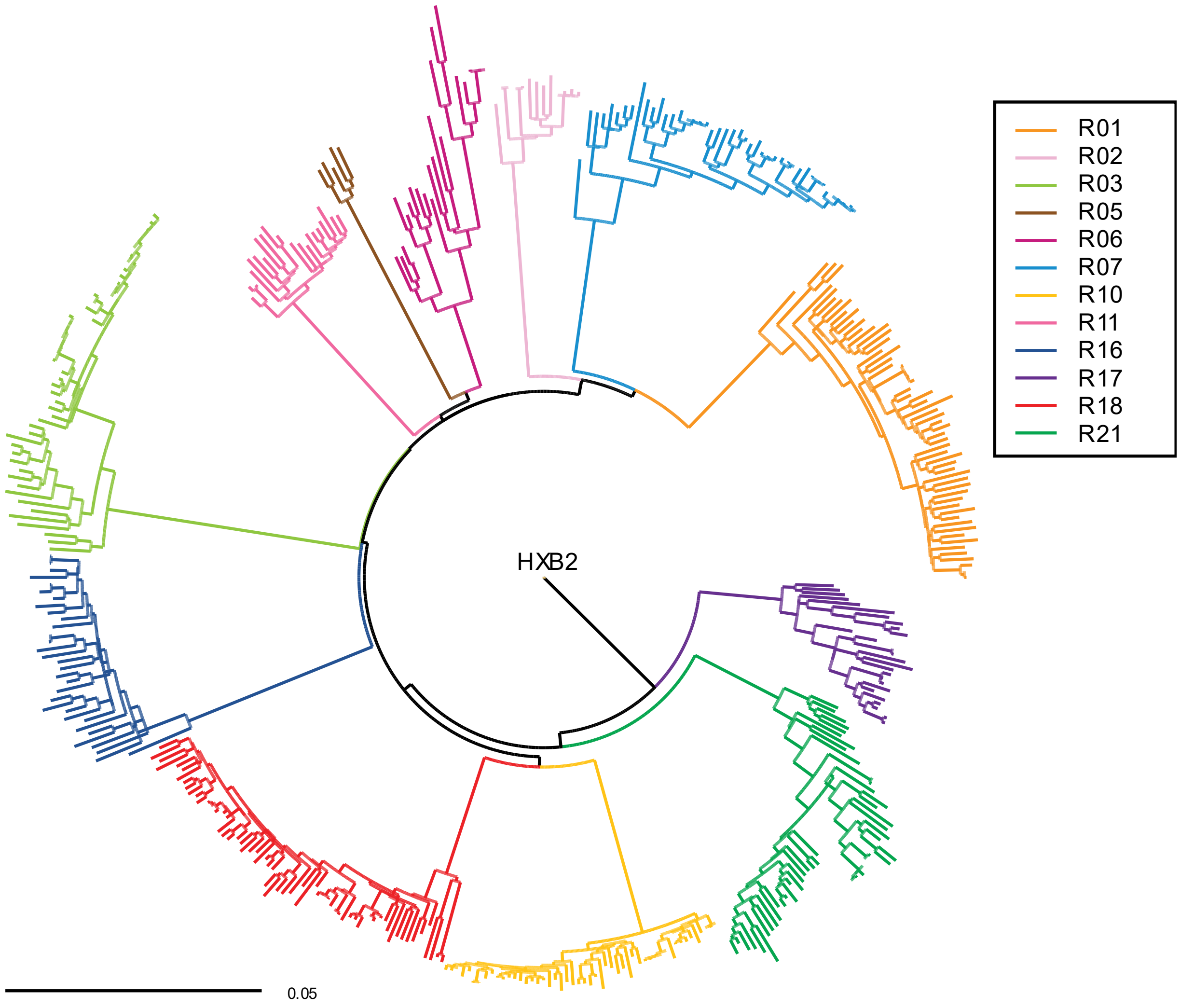

Figure S6. Participant-specific clustering of *env* sequences from positive viral outgrowth wells and proviral sequencing. A neighbor-joining phylogenetic tree of combined *env* sequences from 12 representative participants for whom sequencing was done is shown. The tree was rooted to HXB2. Bar indicates number of nucleotide substitutions per site.

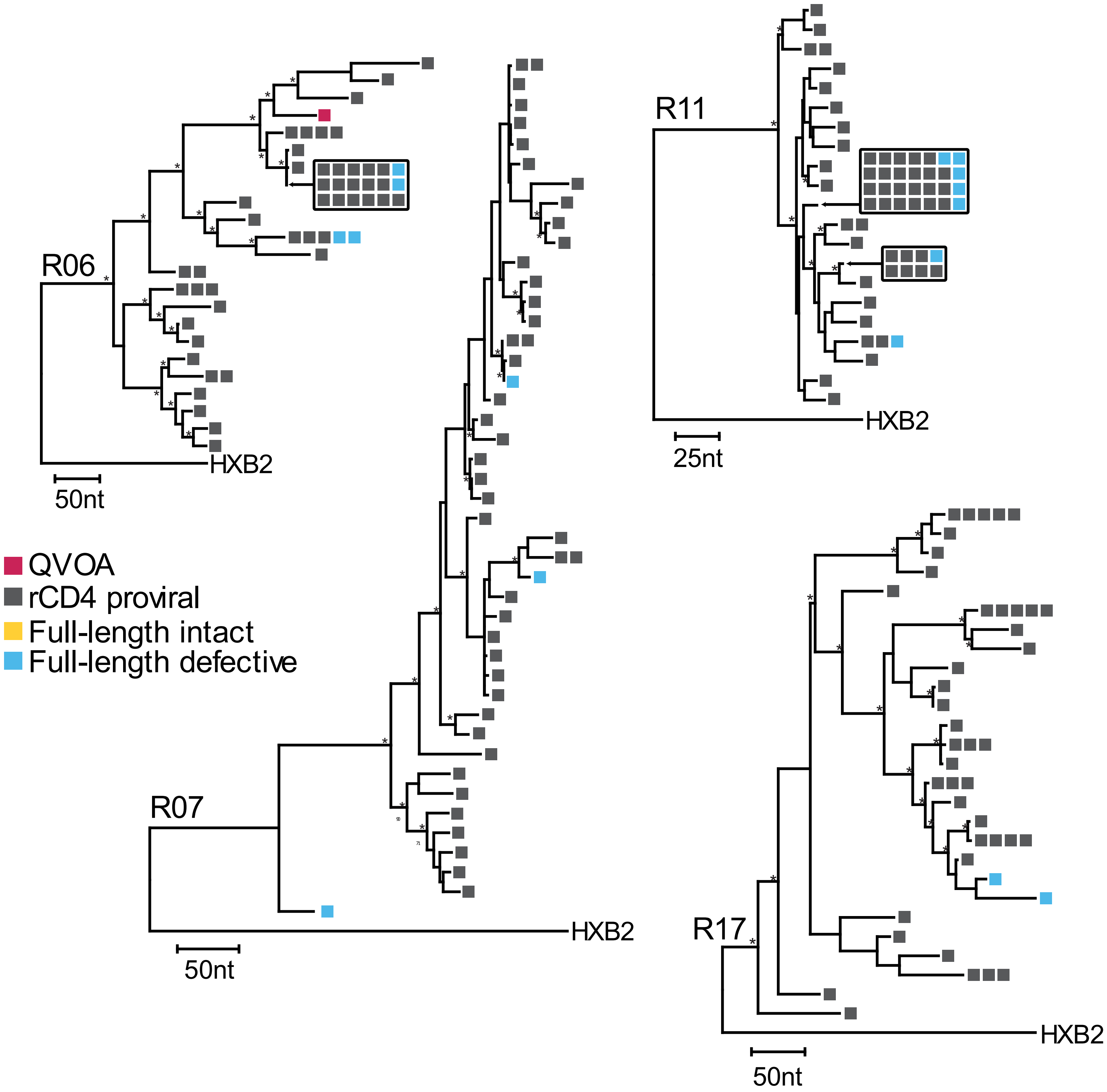

Figure S7. Large expanded clones dominate the population of persistent proviruses in PWH on very long-term ART who have less viral outgrowth. Maximum likelihood phylogenetic trees of *env* sequences for participants not shown in **Figure 5**, each rooted to HXB2. Single genome

sequencing of *env* was performed on cDNA reverse-transcribed from extracted viral RNA from QVOA wells scored positive for viral outgrowth (red) or proviral DNA from resting $CD4^+$ T cells (rCD4, grey). Near full-length genome sequences were obtained using the same proviral DNA and are annotated as intact (yellow) or defective (blue). Bootstrap values greater than 70% are noted by an asterisk. Genetic distance is represented by the scale in nucleotides.

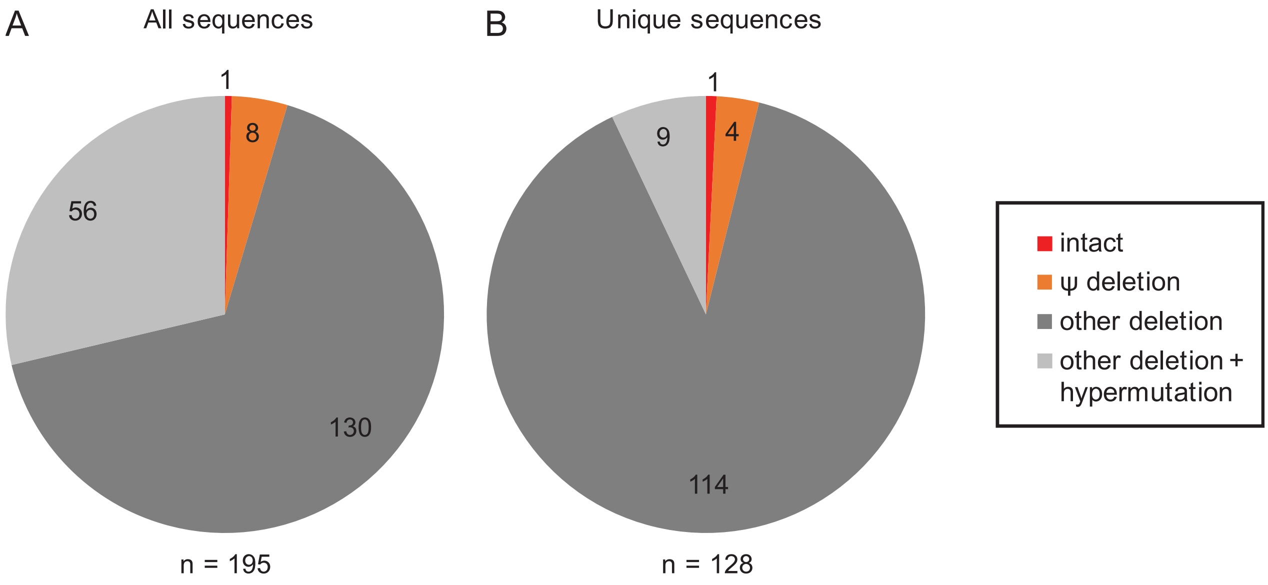

Figure S8. Near full-length sequences of proviruses from PWH on very long-term ART. (**A**) Properties of 195 independent sequences from 9 representative study participants. Sequences were classified as previously described (34, 38, 92) into the following categories: intact (no deletions, hypermutation, or internal stop codons), Ψ deletions (small deletions in the packaging signal region), other deletions, and other deletions plus G→A hypermutation. We did not find any defective proviruses that had internal stop codons as the sole defect. All of the hypermutant proviruses also had deletions. (**B**) Unique sequences from the sequence set in **A**. Independent sequences identical to each other were collapsed into a single sequence to reduce distortions in the landscape resulting from expansion of a single clone.

Table S1. Characteristics of study participants.

ID	Age*	Sex	Race†	Time HIV+ (years)	Current ART regimen‡	Time on Suppressive ART regimen§ (years)	Age at initiation of suppressive ART (yrs)	CD4 nadir (cells/ µl)	Plasma HIV-1 RNA* (copies/ ml)
R01	68	M	AA	25.3	DTG/DOR	21.7	46.3	>200	<20
R02	57	F	AA	22.9	ABC/DTG/3TC	22.8	34.2	320	<20
R03	48	M	AA	30.4	BIC/FTC/TAF	23.6	24.4	316	<20
R04	58	M	W	28.4	ABC/DTG/3TC	17.4	40.6	216	<20
R05	65	M	W	26.1	3TC/DTG/DRV/r	25.9	39.1	214	<20
R06	62	M	AA	30.5	BIC/FTC/TAF	21.4	40.6	18	<20
R07	62	M	AA	29.8	DTG/RPV	19.1	42.9	286	<20
R08	62	M	AA	26.0	DTG/RPV, DRV/c	21.4	40.6	180	<20
R09	68	F	AA	35.8	DTG/RPV, DRV/c	19.9	48.1	64	<20
R10	72	M	W	30.8	BIC/FTC/TAF	22.8	49.2	80	<20
R11	67	F	AA	24.8	BIC/FTC/TAF	22.4	44.6	145	<20
R12	58	F	AA	35.8	BIC/FTC/TAF	24.3	33.7	33	<20
R13	68	F	AA	28.8	ABC/DTG/3TC	22.9	45.1	155	<20
R14	66	M	AA	30.3	RPV/DTG	17.3	48.7	8	<20
R15	63	M	AA	25.8	BIC/FTC/TAF	21.8	41.2	95	<20
R16	56	M	PI	30.8	BIC/FTC/TAF, ECV	25.4	31.7	32	<30
R17	71	M	W	27.4	FTC/TAF, DTG	25.7	45.3	9	<30
R18	67	M	W	36.7	DTG, RPV/TAF/FTC	21.3	45.7	86	<30
R19	57	M	W	19.9	RPV/TAF/FTC	19.8	37.2	200	<30
R20	55	M	AA	23.8	RPV/TDF/FTC	23.8	31.2	25	<30
R21	81	M	W	36.3	BIC/FTC/TAF	26.7	54.3	270	<30
R22	75	M	W	36.7	BIC/FTC/TAF	26.8	48.2	13	<30
R23	68	M	W	34.4	ABC/DTG/3TC	26.0	42.0	120	<30
R24	63	M	AA	20.8	BIC/FTC/TAF	16.8	46.2	185	<20
R25	70	M	W	37.8	RPV/FTC/TAF/EVG/c	25.8	44.2	3	<20
R26	70	M	W	35.3	BIC/FTC/TAF/DOR	21.3	48.7	370	<20
R27	64	M	W	29.8	RAL/DOR/MVC	26.8	37.2	2	<20
R28	56	M	W	28.4	BIC/FTC/TAF/DOR	26.3	29.7	270	<20
R29	60	M	W	34.4	BIC/FTC/TAF	14.9	45.1	107	<20
R30	62	M	W	35.4	BIC/FTC/TAF	26.4	35.6	475	<40
R31	59	M	W	26.4	BIC/FTC/TAF	26.2	32.8	24	<20
R32	57	M	W	20.4	BIC/FTC/TAF	20.3	36.7	329	<40
R33	59	M	W	21.1	ABC/DTG/3TC	21.0	38.0	180	<40
R34	57	M	W	29.8	ABC/DTG/3TC	20.6	36.4	140	<40
R35	58	M	Mixed	22.5	ABC/DTG/3TC	21.7	36.3	20	<40
R36	86	M	W	22.5	ABC/DTG/3TC	17.6	68.4	350	<40
R37	53	M	H	23.4	ABC/DTG/3TC	21	32.0	8	<40
R38	52	M	AS	20.7	ABC/DTG/3TC	20.6	31.4	<200	<40
R39	72	M	W	29.4	BIC/FTC/TAF, DOR	24.2	47.8	10	<40
R40	47	M	W	20.5	BIC/FTC/TAF	20.3	26.7	536	<40
R41	60	M	W	32.7	MVC/DOR/DTG	20.3	39.7	258	<40
R42	65	M	W	30.7	ABC/DTG/3TC	17.9	47.1	<200	<40
Mean	63			28.6		22.1	40.8	161	
Median	62			29.1		21.7	40.6	150	

*At most recent sample date.

†AA, African-America; W, White; H, Hispanic, PI, Pacific Islander; AS, Asian.

‡Abbreviations: 3TC, lamivudine; ABC, abacavir; BIC, bictegravir; /c, cobicistat; DOR, doravirine; DRV, darunavir; DTG, dolutegravir; ECV, entecavir; EVG, elvitegravir; FTC, emtricitabine; MVC, maraviroc; RAL, raltegravir; RPV, rilpivirine; /r, ritonavir; TAF, tenofovir alafenamide; TDF, tenofovir disoproxil fumarate.

§Time on an ART regimen or regimens that maintained continuous suppression of viremia to below the limit of detection through the most recent sampling date (except for isolated blips).

Table S2. Changes in drug regimens during suppressive ART.

ID	Regimen*	Time on suppressive ART (years)
R1	3TC/ZDV/EFV	0.0
	FTC/TFV/EFV	6.3
	FTC/RAL/ATV/RTV	7.5
	DTG/DOR	18.1
R2	d4T/3TC/EFV	0.0
	3TC/ABC/EFV	3.0
	3TC/ABC/NVP	4.3
	3TC/ABC/DTG	16.7
R3	NFV/EFV/d4T/3TC	0.0
	EFV/d4T/3TC	1.3
	EFV/ABC/3TC	5.4
	FTC/RPV/TDF	14.1
	FTC/RPV/TAF	19.2
	BIC/FTC/TAF	20.5
R4	FTC/ddI/ABC/ATV/RTV	0.0
	ABC/3TC/ATV/RTV	4.0
	ABC/3TC/DTG	11.7
R5	3TC/IDV/ZDV	0.0
	3TC/EFV/ABC/ZDV	4.3
	3TC/EFV/ABC/ATV/RTV	8.8
	3TC/DTG/DRV/RTV	18.7
R6	3TC/d4T/EFV	0.0
	FTC/TDF/EFV	3.8
	EVG/COBI/FTC/TAF	16.3
	BIC/FTC/TAF	21.3
R7	RTV/ATV/ddI/TDF	0.0
	LPV/RTV/ddI/TDF	4.6
	RTV/ATV/ddI/TDF	7.3
	RTV/ATV/RAL/TDF	8.1
	RTV/ATV/FTC/TDF	8.2
	RTV/ATV/FTC/ETR	9.1
	ATV/COBI/FTC/TAF	14.0
	DTG/RPV	15.0
R8	LPV/RTV/IDV/EFV	0.0
	DRV/RTV/RAL/EFV	7.4
	DRV/COBI/DTG/RPV	18.0
R9	3TC/AZT/TDF/LPV/RTV	0.0
	LPV/RTV/FTC/TDF/RAL	5.4
	LPV/RTV/ETR/RAL	9.8
	DRV/RTV/ETR/RAL	11.5
	DRV/RTV/FTC/TDF	12.8
	DTG/RPV/3TC/TAF	13.8
	DRV/COBI	15.9
R10	RTV/3TC/ABC	0.0
	LPV/RTV/3TC/ABC/TDF	4.1
	TDF/RAL/ETR	9.2
	3TC/ABC/RAL/ETR	12.3
	BIC/FTC/TAF	18.5
R11	3TC/d4T/EFV	0.0
	3TC/EFV/TDF/ABC	2.9
	3TC/EFV/TDF	3.5
	FTC/TDF/EFV	4.7
	ABC/DTG/3TC	14.6
	BIC/FTC/TAF	17.8
R12	RTV/SQV/EFV/ddI	0.0
	ddI/EFV	0.8
	ddI/EFV/RTV/IDV	1.2
	ddI/LPV/RTV/NVP	2.2
	LPV/RTV/FTC/TDF/NVP	6.8
	EFV/FTC/TDF/LPV/RTV	10.4

	RTV/DRV/EFV/FTC/TDF	12.3
	FTC/TAF/EVG/COBI	17.7
	FTC/TAF/EVG/COBI/DRV	21.3
	FTC/TAF/EVG/COBI	19.1
	BIC/FTC/TAF	20.2
R13	NFV/d4T/3TC	0.0
	ABC/3TC/AZT/NFV	2.4
	3TC/AZT/EFV	2.8
	EFV/FTC/TDF	8.9
	ABC/DTG/3TC	16.0
R14	LPV/RTV/ABC/3TC/AZT/TDF/EFV	0.0
	LPV/RTV/ABC/3TC/AZT/TDF	1.1
	DRV/RTV/RAL/ETR	2.9
	DTG/RPV	15.8
R15	3TC/AZT/NFV	0.0
	3TC/AZT/NFV/ABC	0.2
	ABC/3TC/AZT/TDF/NFV	1.0
	ABC/3TC/AZT/TDF/ATV/RTV	2.9
	FTC/TDF/ATV/RTV	7.9
	FTC/TAF/EVG/COBI	15.2
	BIC/FTC/TAF	18.7
R16	D4T/3TC/EFV/RTV/FTV/SQV	0.0
	ABC/3TC/EFV/RTV/SQV	4.0
	ABC/3TC/EFV/LPV/RTV	4.3
	FTC/TDF/EFV/LPV/RTV	11.6
	FTC/TDF/EFV	13.1
	EVG/COBI/FTC/TAF	20.4
	BIC/FTC/TAF	21.0
R17	D4T/3TC/IDV	0.0
	3TC/TDF/ATV/RTV	10.1
	ABC/3TC/ATV/RTV	14.4
	ABC/DTG/3TC	19.5
	FTC/TAF/DTG	22.6
R18	DDI/EFV/TDF/FTC	7.8
	EFV/TDF/FTC/RAL	9.1
	RPV/TAF/FTC/DTG	15.8
R19	EFV/AZT/3TC	0.0
	EFV/FTC/TDF	4.3
	RPV/TDF/FTC	12.2
	RPV/TAF/FTC	16.5
R20	ABC/D4T/3TC/EFV	0.0
	ABC/3TC/EFV	1.9
	RPV/TDF/FTC	15.0
R21	ABC/D4T/3TC/TDF/EFV	8.0
	ABC/3TC/TDF/EFV	8.6
	EFV/TDF/FTC	13.3
	FTC/ABC/3TC/EFV	16.6
	ABC/3TC/EFV	17.2
	EFV/TDF/FTC	18.1
	BIC/FTC/TAF	26.7
R22	ABC/3TC/IDV	0.0
	AZT/3TC/ABC	7.8
	ABC/3TC/TDF	8.1
	AZT/TDF/ABC/3TC	8.9
	ABC/3TC/ATV/RTV	9.5
	ABC/3TC/ATV	10.5
	ABC/3TC/RAL	16.6
	ABC/DTG/3TC	20.5
	BIC/FTC/TAF	26.7
R23	ABC/D4T/NFV	0.0
	TDF/ABC/3TC/ATV/RTV	8.7
	TDF/ABC/3TC/RTV/DRV	15.1
	ABC/3TC/RTV/DRV	17.3
	ABC/DTG/3TC	18.3
	FTC/TAF/DTG	25.7
	ABC/DTG/3TC	26.0

R24	ATV	0.0
	ATV/RTV	3.8
	ATV	4.0
	3TC/AZT/ATV	8.1
	ATV/FTC/TAF	12.3
	BIC/FTC/TAF	13.2
R25	3TC/IDV/d4T	0.0
	3TC/SQV/RTV/d4T	2.5
	SQV/RTV/NVP/ABC	3.5
	LPV/RTV/NVP/ABC	5.9
	SQV/LPV/RTV/NVP/ABC	8.4
	RTV/DRV/FTC/TDF/NVP	12.2
	ETR/RAL/RTV/DRV	14.6
	FTC/TAF/EVG/COBI/RPV	18.9
R26	3TC/LPV/RTV/d4T	0.0
	LPV/RTV/d4T	0.1
	LPV/RTV/d4T/ABC	2.4
	LPV/RTV/d4T	2.6
	RTV/TDF	3.1
	RTV/ATV/TDF	3.2
	ATV/d4T	3.3
	3TC/RTV/ATV/TDF	3.3
	3TC/EFV/TDF	4.3
	3TC/NVP/TDF	6.3
	FTC/TDF/NVP	7.7
	3TC/NVP/TDF	8.1
	FTC/TDF/NVP	8.7
	MVC/ETR/RAL	11.8
	FTC/TAF/EVG/COBI/DRV	15.7
	BIC/FTC/TAF/DOR	18.3
R27	SQV/LPV/RTV/EFV/ABC/3TC/AZT	10.3
	MVC/RAL/LPV/RTV/ABC/TDF	12.4
	MVC/RAL/ABC/RTV/DRV/TDF	16.4
	MVC/RAL/ABC/RTV/DRV	16.7
	MVC/RAL/ABC/RTV/DRV	19.6
	FTC/TAF/RPV/DTG	20.8
	RPV/FTC/TAF/EVG/COBI	21.0
	FTC/TAF/RPV/RAL	22.0
	FTC/TAF/RAL	22.3
	DTG/RPV	22.8
	DTG/RPV/RTV/DRV	23.0
	DTG/RPV	23.0
	FTC/TAF/DTG/RPV	23.2
	MVC/DRV/COBI	23.6
	3TC/DTG/DOR	23.8
	RALHD/DOR	24.5
	MVC/RALHD/DOR	25.8
R28	3TC/AZT	10.3
	LPV/RTV/FTC/TDF	16.8
	LPV/RTV/FTC/TDF/EVG/COBI	17.8
	FTC/TDF/EVG/COBI	17.8
	ETR/FTC/TDF/EVG/COBI	18.8
	RPV/DRV/COBI/DTG	19.1
	RPV/RTV/DRV/COBI/DTG	19.4
	RPV/RTV/DRV/DTG	20.3
	RPV/DTG	20.6
	RPV/DRV/COBI/DTG	21.4
	RPV/RTV/DRV/DTG	21.9
	RPV/FTC/TAF/EVG/COBI	22.1
	BIC/FTC/TAF/DOR	26.1
R29	LPV/RTV/ABC	0.0
	LPV/RTV/FTC/TDF	0.9
	FTC/TDF/NVP	1.0
	ABC/NVP	4.3
	BIC/FTC/TAF	11.8
R30	3TC/SQV/AZT	0.0

	3TC/IDV/AZT	0.4
	3TC/IDV/RTV/AZT	3.4
	3TC/AZT/LPV/RTV	7.5
	3TC/AZT/LPV/RTV/TDF	11.0
	LPV/RTV/FTC/TDF	11.1
	MVC/RAL/LPV/RTV/FTC/TDF	11.6
	LPV/RTV/FTC/TDF	12.6
	RTV/DRV/FTC/TDF	16.5
	MVC/ETR/RAL	19.6
	FTC/TAF/DTG	20.4
	FTC/TAF/EVG/COBI	21.5
R31	3TC/SQV/AZT	0.0
	3TC/IDV/AZT	0.3
	3TC/AZT/EFV	3.8
	3TC/AZT/NVP	5.3
	3TC/NVP/ABC	7.7
	ABC/NVP	9.8
	BIC/FTC/TAF	25.8
R32	3TC/AZT/LPV/RTV	0.0
	LPV/RTV/ABC	0.0
	NFV/ABC	0.1
	DLV/NFV	3.0
	LPV/RTV	4.2
	LPV/RTV/FTC/TDF	4.9
	RAL/LPV/RTV/FTC/TDF	5.3
	MVC/RAL/LPV/RTV/FTC/TDF	5.7
	RAL/LPV/RTV/FTC/TDF	7.0
	3TC/MVC/RAL/LPV/RTV	9.2
	FTC/TAF/EVG/COBI	14.3
	BIC/FTC/TAF	17.1
R33	3TC/AZT/LPV/RTV/ABC	0.0
	LPV/RTV/ABC/3TC/AZT	0.6
	3TC/RTV/ATV/ABC	2.8
	ABC/RTV/ATV	3.9
	LPV/RTV/ABC	5.6
	LPV/RTV/FTC/TDF	7.1
	ETR/ABC	8.8
	ABC/DTG	12.1
	ABC/DTG/3TC	13.1
R34	3TC/IDV/AZT	0.0
	3TC/AZT/LPV/RTV	0.2
	3TC/AZT/LPV/RTV/ABC	0.3
	LPV/RTV/ABC/3TC/AZT	0.3
	3TC/RTV/ATV/ABC	2.2
	ABC/RTV/ATV	3.7
	LPV/RTV/ABC	4.8
	MVC/RAL/LPV/RTV/ABC	6.0
	MVC/RAL/ABC/RTV/DRV	10.6
	MVC/RAL/ABC	10.8
	ABC/DTG/3TC	12.7
	BIC/FTC/TAF	16.5
	ABC/DTG/3TC	16.5
	BIC/FTC/TAF	16.8
	ABC/DTG/3TC	17.0
R35	3TC/AZT/LPV/RTV	0.0
	3TC/LPV/RTV/TDF	2.4
	3TC/LPV/RTV/ABC	3.3
	3TC/RTV/ATV/ABC	3.5
	ABC/RTV/ATV	5.8
	EFV/FTC/TDF	8.3
	ABC/EFV	12.0
	ABC/DTG	12.8
	ABC/DTG/3TC	13.9
R36	3TC/BLINDED TRIAL/TDF	0.0
	LPV/RTV/FTC/TDF	2.6
	RAL/FTC/TDF	2.8

	RAL/ABC	8.3
	ABC/DTG	9.2
	ABC/DTG/3TC	10.3
R37	3TC/AZT/EFV/ABC	0.0
	ABC/EFV	4.6
	EFV/FTC/TDF	6.8
	ABC/DTG/3TC	13.2
R38	3TC/AZT/EFV	0.0
	3TC/EFV/ABC	1.8
	ABC/EFV	4.3
	EFV/FTC/TDF	6.3
	ABC/EFV	9.3
	ABC/DTG	12.6
	ABC/DTG/3TC	12.9
R39	EFV/ddI/NFV/d4T/ABC	0.0
	LPV/RTV/EFV/ddI/ABC	2.1
	LPV/RTV/EFV/TDF/ABC	3.6
	3TC/RTV/ATV/EFV/ABC	5.6
	ABC/RTV/ATV/EFV	7.3
	LPV/RTV/ABC/EFV	9.7
	LPV/RTV/EFV/FTC/TDF	10.0
	RAL/LPV/RTV/EFV	11.1
	RPV/DRV/COBI/DTG	16.2
	RPV/RTV/DRV/DTG	16.9
	RPV/DRV/COBI/DTG	18.0
	BIC/FTC/TAF/DOR	22.6
R40	3TC/AZT/LPV/RTV	0.0
	3TC/AZT/EFV	1.6
	3TC/EFV/ABC	1.7
	ABC/EFV	3.8
	EFV/FTC/TDF	6.4
	FTC/TAF/EVG/COBI	13.9
	BIC/FTC/TAF	18.8
R41	3TC/LPV/RTV/EFV/TDF/ABC	0.0
	ABC/RTV/ATV/EFV/TDF	3.7
	ETR/ABC/RTV/DRV	7.6
	ETR/RAL/ABC/RTV/DRV	7.9
	RPV/DRV/COBI/ABC/DTG/3TC	12.4
	DOR/DRV/COBI/ABC/DTG/3TC	17.2
	MVC/DOR/DTG	19.8
R42	3TC/AZT/NFV	0.0
	3TC/AZT/DLV	1.0
	ETR/ABC	5.2
	ABC/DTG/3TC	10.6

*Changes in drug regimens during suppressive ART. For drug abbreviations, see **Table S1**.

Table S3. Blips during suppressive ART

ID	Blips >100 (c/ml)	Time on suppressive ART (yrs)	Assay LOD* (c/ml)	Previous HIV-1 RNA† (c/ml)	Time on suppressive ART (yrs)	Next HIV-1 RNA‡ (c/ml)	Time on suppressive ART (yrs)	Time since blip (days)
R2	228	8.69	50	<400	8.59	<50	9.22	196
	121	21.50	20	<20	21.36	<20	21.71	75
R3	113	3.38	50	16	3.21	11	3.40	6
	126	5.41	50	<50	5.14	<50	5.42	3
R6	160	15.61	20	<20	15.00	<20	15.98	133
R8	109	11.24	50	<50	10.42	<20	11.30	21
R10	280	2.50	50	148	0.25	80	4.15	602
R11	158	1.72	50	25	1.04	177	1.99	100
	177	1.99	50	158	1.72	64	2.41	154
R12	186	0.88	50	11	0.64	171	1.04	57
	171	1.04	50	186	0.88	16	1.12	29
	195	3.07	50	24	3.02	16	3.27	72
	3851	10.85	50	<50	10.63	<50	10.87	7
R13	1030	0.80	50	13	0.61	17	0.84	13
	219	1.18	50	21	0.90	137	1.41	84
	137	1.41	50	219	1.18	19	1.53	44
R15	119	2.95	50	<50	2.72	<50	3.13	63
R16	138	1.92	50	<50	1.69	<50	1.96	14
R20	104	3.19	50	<50	3.10	<50	3.39	73
	319	5.03	75	<75	4.82	<75	5.25	80
R22	145	6.92	75	<75	6.71	<50	7.00	29
R23	15228	3.36	50	<50	3.08	<50	3.41	18
	103	6.11	75	<75	5.78	<75	6.28	62
R25	115	1.58	500/50	<500	1.33	<50	1.92	124
R29	102	10.36	40	<40	9.86	<40	10.41	18
R30	101	6.84	50	<50	6.67	<50	6.87	11
R31	179	9.59	50	<50	9.18	<50	9.79	73

*Limit of detection of the assay for plasma HIV-1 RNA used at the time of the blip.

†HIV-1 RNA measurement at visit prior to the blip.

‡HIV-1 RNA measurement at visit after to the blip.

Table S4. Decay of cells with inducible, replication-competent proviruses as measured by QVOA.

Time on ART* (yrs)	Donors studied	Fraction of donors with outgrowth (%)	Total assays	Fraction of assays with outgrowth (%)	Positive assays		All assays†	
					Geometric mean frequency (IUPM)	Mean time on ART (yrs)	Geometric mean frequency (IUPM)	Mean time on ART (yrs)
<7‡	62	95.2	171	86.5	0.54	3.1	0.44	3.0
>7	42	90.4	61	88.5	0.62	20.5	0.46	20.6

*Time from the start of a continuously suppressive ART regimen. Isolated blips were not considered an exclusion.

†For negative assays, the maximum value based on the number of cells plated was used.

‡Data from Siliciano et al., 2003 (reference 29).

Table S5. Models for decay of inducible, replication-competent HIV-1 with very long-term ART.

Model	Model constraints*	Initial decay $t_{½}$ (mo)	Initial decay 95% CI (mo)	t_e (yrs)†	2nd phase‡ t_2 (yrs)	2nd phase‡ 95% CI in yrs t_2 ($t_{½}$)	-LL§	BICc¶
simple exponential		89 yr	18 yr to ∞				425	450
segmented exponential		21	12–110	2.9	60	15 to ∞ (29 to ∞)	410	451
	initial $t_{½}$ = 44 mo	44		3.1	97	18 to ∞ (27 to ∞)	414	450
	t_e = 7 yr	55	31–278	7	20	8 to ∞ (41 to ∞)	419	455
	t_e = 7 yr, initial $t_{½}$ = 44 mo	44		7	23	10 to ∞ (73 to ∞)	419	449
biexponential		6	4–13		28	12 to ∞ (69 to ∞)	408	454

*QVOA data in **Figure 3B** were modeled using simple exponential decay, segmented exponential decay, and biexponential decay (also **Figure S5**). If a model constraint is indicated, then the corresponding parameter was fixed and not fitted. The fit in **Figure 3B** corresponds to the selected model shown in the next to last row in the Table with the initial phase $t_{1/2}$ = 44 months, a breakpoint at 7 year and t_2 = 23 years

†Time at which the decay rate changes.

‡After the initial decay for time t_e, the segmented models and the biexponential model estimated a slow increase in the levels of cells with inducible, replication-competent HIV-1. This rate of increase is expressed as a doubling time (t_2). However, the 95% CI on the second phase is very wide and includes in all cases the possibility of a constant level or even a slow decrease. Thus, in the Table we include two rows which together represent the 95% CI for the second phase. The first row indicates the limits of the CI when the levels are increasing and the second row, in parentheses, the limits when the levels are decreasing. Thus, for our selected model shown in the next to last row in the Table with the initial phase $t_{1/2}$ = 44 months, a breakpoint at 7 year

and t_2 = 23 years, the 95% CI of the second phase includes a doubling time from 10 years to infinity (i.e. no increase), and a half-life from 73 years to infinity (i.e. no decay).

§Log likelihood.

¶Bayesian information criterion corrected.

Table S6. Best fit models for decay of intact proviruses as measured by IPDA.

Model	Model constraints*	Initial decay		t_e (yrs)	2nd phase†		-LL‡	BICc§
		$t_{½}$ (mo)	95% CI (mo)		$t_{½}$ (yrs)	95% CI (yrs)		
simple exponential		68	57-84				418	444
segmented exponential	t_e = 6 yr	40	30-62	6	10	7-18	406	443
	t_e = 7 yr	46	34-70	7	9	6-18	407	444
biexponential		12	6-17		10	6-17	413	459

*IPDA data in **Figure 3C** were modeled using simple exponential decay, segmented exponential decay, and biexponential decay. The best fits were obtained with segmented exponential decays. We varied the time at which the decay rate changed (t_e), from 2 to 12, and got the best fits with t_e = 6 or 7 yrs. The fit in **Figure 3C** corresponds to the next to last row in the Table with the initial phase $t_{1/2}$ = 46 months, a breakpoint at 7 year and a second phase $t_{1/2}$ = 9 years.

†After the initial decay, the best fit models all showed a second phase with a slower decay rate.

‡Log likelihood.

§Bayesian information criterion corrected.

Table S7. Best fit models for decay of defective proviruses as measured by IPDA.

Provirus type	Model*	Model constraints	Initial decay		t_e (yrs)	2nd phase		-LL‡	BICc§
			$t_{½}$ (yrs)	95% CI (yrs)		$t_{½}$ (yrs)	95% CI (yrs)		
3′del/HM	*simple exponential*		6	14-26				228	257
	segmented exponential	t_e = 9 yr	16	11-34	9	42	17 to ∞ (81 to ∞)	221	257
	biexponential		8	5-23		20	11 to ∞ (65 to ∞)	268	312
5′del/HM	*simple exponential*	NA	25	16-18	NA	NA	NA	302	328
	segmented exponential	t_e = 9 yr	38	14 to ∞ (49 to ∞)	9	27	12 to ∞ (124 to ∞)	302	338
	biexponential		20	2 to ∞ (3 to ∞)		35	12 to ∞ (43 to ∞)	337	384

*IPDA data in **Figure 2D** were modeled using simple exponential decay, segmented exponential decay, and biexponential decay. The best fits were obtained with segmented exponential decay for 3′del/HM (3' deleted/hypermutated) proviruses and linear decay for 5′del (5' deleted) proviruses. Entries in red refer to doubling times and corresponding limits of the confidence intervals. When the upper bound is infinite, it implies no increase or decrease.

‡Log likelihood.

§Bayesian information criterion corrected.
